# Bioavailability of Orally Administered rhGM-CSF: A Single-Dose, Randomized, Open-Label, Two-Period Crossover Trial

**DOI:** 10.1371/journal.pone.0005353

**Published:** 2009-05-12

**Authors:** Wenping Zhang, Zhengbing Lv, Zuoming Nie, Guogang Chen, Jian Chen, Qing Sheng, Wei Yu, Yongfeng Jin, Xiangfu Wu, Yaozhou Zhang

**Affiliations:** 1 Institute of Biochemistry, College of Life Sciences, Zhejiang University, Hangzhou, China; 2 The Key Laboratory of Bioreactor and Biopharmacy of Zhejiang Province, Institute of Biochemistry, Zhejiang Sci-Tech University, Hangzhou, China; 3 Zhejiang Chinagene Biopharmaceutical Co., Ltd., Haining, China; 4 Institute of Biochemistry, the Chinese Academy of Sciences, Shanghai, China; Bauer Research Foundation, United States of America

## Abstract

**Background:**

Recombinant human granulocyte-macrophage colony-stimulating factor (rhGM-CSF) is usually administered by injection, and its oral administration in a clinical setting has been not yet reported. Here we demonstrate the bioavailability of orally administered rhGM-CSF in healthy volunteers. The rhGM-CSF was expressed in *Bombyx mori* expression system (BmrhGM-CSF).

**Methods and Findings:**

Using a single-dose, randomized, open-label, two-period crossover clinical trial design, 19 healthy volunteers were orally administered with BmrhGM-CSF (8 µg/kg) and subcutaneously injected with rhGM-CSF (3.75 µg/kg) respectively. Serum samples were drawn at 0.0h, 0.5h ,0.75h,1.0h,1.5h,2.0h ,3.0h,4.0h,5.0h,6.0h,8.0h,10.0h and 12.0h after administrations. The hGM-CSF serum concentrations were determined by ELISA. The AUC was calculated using the trapezoid method. The relative bioavailability of BmrhGM-CSF was determined according to the AUC ratio of both orally administered and subcutaneously injected rhGM-CSF. Three volunteers were randomly selected from 15 orally administrated subjects with ELISA detectable values. Their serum samples at the 0.0h, 1.0h, 2.0h, 3.0h and 4.0h after the administrations were analyzed by Q-Trap MS/MS TOF. The different peaks were revealed by the spectrogram profile comparison of the 1.0h, 2.0h, 3.0h and 4.0h samples with that of the 0.0h sample, and further analyzed using both Enhanced Product Ion (EPI) scanning and Peptide Mass Fingerprinting Analysis. The rhGM-CSF was detected in the serum samples from 15 of 19 volunteers administrated with BmrhGM-CSF. Its bioavailability was observed at an average of 1.0%, with the highest of 3.1%. The rhGM-CSF peptide sequences in the serum samples were detected by MS analysis, and their sizes ranging from 2,039 to 7,336 Da.

**Conclusions:**

The results demonstrated that the oral administered BmrhGM-CSF was absorbed into the blood. This study provides an approach for an oral administration of rhGM-CSF protein in clinical settings.

**Trial Registration:**

www.chictr.org ChiCTR-TRC-00000107

## Introduction

Recombinant human granulocyte-macrophage colony-stimulating factor (rhGM-CSF) acts on precursor cell proliferation in bone marrow. It also stimulates granulocytes, monocytes, and colony formation; and induces hyperplasy of macrophages [1] . This protein is primarily used in bone marrow transplantation, tumor chemotherapy, and the treatment of aplastic anemia and agranulocytosis related to AIDS [2]–[6]. The rhGM-CSF is an acidoglycoprotein containing 127 amino acid residues and has a molecular weight of 14.4 kDa to 32 kDa [7]. It can be expressed in *Escherichia coli*, yeast, and mammalian cells [8], [9]. Currently, the rhGM-CSF is administered by injection in clinical settings.

We expressed hGM-CSF in silkworm pupae bioreactor named as BmrhGM-CSF. It was purified and determined to have molecular weigh of 29 kDa [7], [10], [11]. It's post-translational modifications were characterized in addition to its glycosylation [7], [10], [11]., The oral formulation of BmrhGM-CSF was used in our previous animal study. The results from preclinical studies demonstrated that orally administered BmrhGM-CSF could function as an active cytokine [10]. The orally administered BmrhGM-CSF was shown to be absorbed into blood of mice, beagles, and macaques, which increased leukocyte counts. (1) In hematogenesis-inhibited mice, orally administered BmrhGM-CSF stimulated the colony formation in hemopoietic tissues on the spleen surface and DNA synthesis in the bone marrow. The effect was similar to that of injected Leucomax. (2) Hematogenesis-inhibited beagles treated with ^60^Co and orally administered BmrhGM-CSF had white blood cell counts reached 50% of their baseline values by 9^th^ day, and recovered to the normal levels by 12th day. Moreover, the percentage of new granulocytes and promyelocytes in the bone marrow increased significantly. (3) The oral administration of BmrhGM-CSF to cyclophosphamide-treated macaques increased leukocyte production in a manner similar to that of Leucomax injections [10].

BmrhGM-CSF also promoted the production of granulocytes in macaques and beagles, and increased leukocyte counts in a dose-dependent manner. Western blotting analysis indicated that BmrhGM-CSF could be absorbed into blood through the intestinal parva [10].

The results from preclinical animal stuies have proven that BmrhGM-CSF could penetrate into the blood as an active cytokine through the oral administration [10]. In this paper, we report the results of the bioavailability of the orally administered BmrhGM-CSF in a clinical trial.

## Methods

The protocol for this clinical trial and supporting CONSORT checklist are available as supporting information; see Protocol S1 and Checklist S1.

### Participants

A total of 22 male volunteers were screened for inclusion at 2nd Affiliated Hospital of Zhejiang University, School of Medicine (Hangzhou, China). Participants were considered eligible if they met the following criteria:

1. apparently healthy;
2. 18–40 years of age;
3. no liver, kidney, gastrointestinal and metabolic disorders, or neuropsychiatric diseases;
4. no medical history, drug allergies history or smoking or alcoholic drinking;
5. 18 kg/m^2^≤BMI≤25 kg/m^2^;
6. with good communication between researchers, to comply with the requirements of the entire study and
7. with signed informed consent and conducted a comprehensive history and physical examination and laboratory tests including blood pressure, heart rate and body temperature measurements, blood examination, urine analysis, blood biochemistry and ECG, etc.

Exclusion criteria were:

1. participating in other clinical trials in last 2 weeks;
2. donating blood during the trial period or within last 1 month;
3. not accepting physical examination;
4. drug allergy or a history of allergic disease;
5. history of drug abuse and
6. with any chronic or acute disease.

### The test and reference formulations

The test formulation was the BmrhGM-CSF capsule for the oral administration provided by Zhejiang Chinagene Biopharmaceutical Co., Ltd. Each capsule contained 60 µg of BmrhGM-CSF. The reference formulation was the rhGM-CSF for subcutaneous injection (150 µg/bottle) purchased from Guangzhou Baidi Biotechnology Co., Ltd .The rhGM-CSF for subcutaneous injection was expressed in *E. coli.* The subcutaneous injection site was at upper arm deltoid. Both the test and reference formulations were stored at 4°C.

### Ethics

Phase I trial was registered with http://www.chictr.org (ChiCTR-TRC-00000107). We conducted the study in accordance with good clinical practice guidelines, provisions of the Declaration of Helsinki, and regulations of the People's Republic of China. The protocol and consent forms were approved by the ethics committee of 2nd Affiliated Hospital of Zhejiang University, School of Medicine. The Data and Safety Monitoring Board monitored adverse events and confirmed the end points of the experiments if investigators observed adverse events. The written informed consent was obtained from all volunteers.

### Interventions

**Figure 1:**
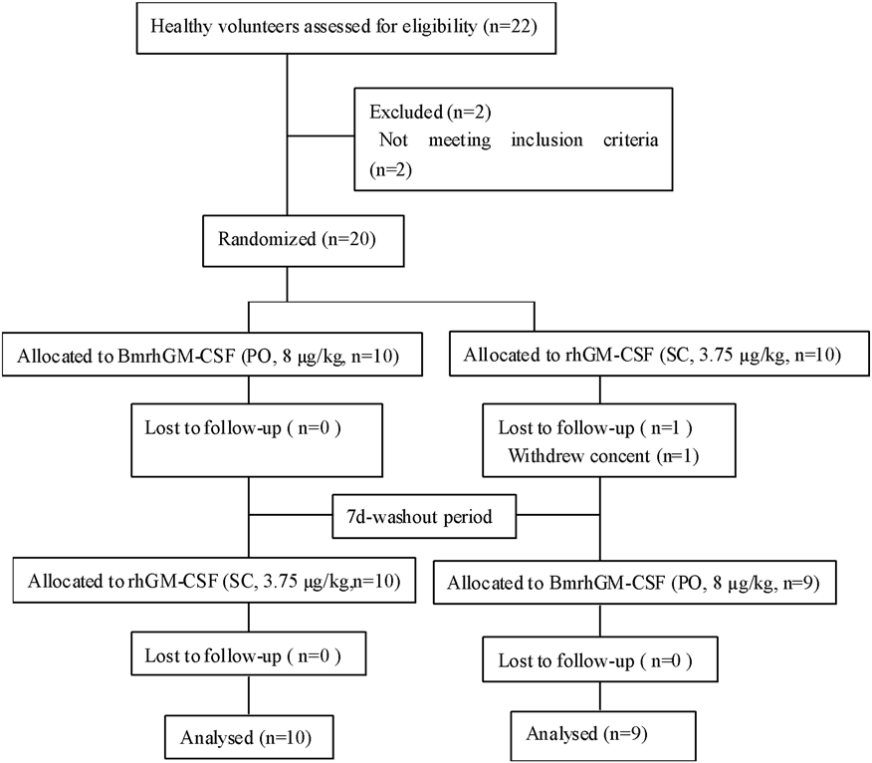
Flow Diagram of Participant Progress through the Study. The test formulation: BmrhGM-CSF (PO: per os); the reference formulation: rhGM-CSF (SC: subcutaneous).

In a single-dose, randomized, open-label, two-period crossover clinical trial, 20 volunteers were divided into two groups, 10 for each. These groups were designated TR (Test Formulation/Reference Formulation) and RT (Reference Formulation/Test Formulation), respectively. Of which, 10 volunteers in the TR group was orally administered with BmrhGM-CSF (8 µg/kg) on Day 1. After a 7-day washout period, the subcutaneous injection of the reference formulation, rhGM-CSF (3.75 µg/kg) was given to each subject of the same TR group on Day 9. The same protocol was performed, vice versa, for the RT group as described in the participant flow chart (Figure 1). After fasting overnight for at least 10 h till 8:00 am on the following day, the subjects designated for the oral administration were given BmrhGM-CSF with 200 ml warm boiled water. The subjects designated for the subcutaneous injection were given the injection at the upper arm deltoid with rhGM-CSF. Followed by a 4-h period, all of the volunteers in both groups had the same low-fat meal. During the trial study, the subjects did not smoke or drink alcohol, caffeine-containing beverage, or fruit juice in order to avoid any possible interference on the absorption and metabolism of the drug. The administration order for the subjects depended on random drawn numbers. The administration intervals between subjects depended on the length of time required for withdrawing blood samples for 2 to 3 min.

### Objectives

The primary goals of the study were to: (1) investigate the bioavailability of orally administered BmrhGM-CSF in healthy volunteers; (2) demonstrate the presence of rhGM-CSF peptides in serum of volunteers orally administered with BmrhGM-CSF.

### Outcomes

#### The hGM-CSF concentrations in the blood serum

Blood (2 ml) was collected from the brachial vein with a single-use syringe after drug administration at 0.0h, 0.5h, 0.75h, 1.0h, 1.5h, 2.0h, 3.0h, 4.0h, 5.0h, 6.0h, 8.0h, 10.0h and 12.0h. The blood samples were drawn into EDTA-coated anticoagulation tubes, mixed thoroughly, and centrifuged at 3000 rpm. Then serum samples were collected and stored at 4°C for use. The hGM-CSF concentrations in the sera were determined by ELISA within 12h after collection. Human GM-CSF ELISA kits were purchased from Jingmei Bioengineering Co., Ltd (Beijing, China). In order to reduce pilot error, these human GM-CSF ELISA kits used were the same lot. The tests were performed according to the instruction of the ELISA kit. Briefly, a standard curve was generated as follows: a solution containing 2000 ng/L of hGM-CSF was prepared, and standard curve calibrators containing 7.8, 15.6, 31.2, 62.5, 125.0, 250.0, 500.0, and 1000.0 ng/L of hGM-CSF were generated by a serial dilution. The OD values of the standard curve calibrators, quality controls and samples tested were determined at a wavelength of 450 nm. The hGM-CSF concentrations of the tested samples were measured by the regression analysis with the CVXPT32 software.

#### MS Analysis

The serum samples collected from 3 volunteers at the 0.0h, 1.0h, 2.0h, 3.0h , 4.0h after the oral administration of BmrhGM-CSF and the subcutaneous injection of rhGM-CSF were subjected to MS analysis (Q-Trap MS/MS TOF, ABI Co.) . Those 3 were randomly selected from 15 ELISA detectable subjects. The serum samples were diluted by 100-fold with 80% acetonitrile and the solution was centrifuged at 12,000 rpm for 10 min. The supernatant was collected for MS analysis. The 0.0h sample analysis spectrogram was used as control and compared with the analysis spectrogram of 1.0h, 2.0h, 3.0h and 4.0h hour sample in order to obtain peaks with different mass-to-electric charge ratios. Further analysis of the peaks through both Enhanced Product Ion (EPI) scanning and Peptide Mass Fingerprinting Analysis concluded that the molecular weight and amino acid sequences of these peptides matching hGM-CSF fragments.

#### Randomization

In a single-dose, randomized, open-label, two-period crossover clinical trial, 20 enrolled participants were randomly assigned to two groups (TR and RT) in a 1∶1 ratio. Briefly, a randomization code was developed using a computer-generated randomization schedule (Compaq Visual Fortran version 6.5, IMSL Fortran Library, Compaq Computer Corp., Houston, Texas) by a biostatistician. After preparing a random sequence, subjects were allocated to the two trial groups by a physician who was not involved in the main research. During the process of allocation, each subject was given a unique identification code (Unique Identifier, UI). This UI was used as a label to uniquely identify the subject's group after completion of the study. During the study period this UI was given to the main researchers together with the necessary treatment for each participant. Both test and reference formulations of the rhGM-CSF were designed for either oral or subcutaneous administration in both RT and TR groups. Specifically, the test formulation of BmrhGM-CSF was orally administered to the TR group on Day 1. After a 7-day washout period, the reference formulation of rhGM-CSF was subcutaneously injected on Day 9. Conversely, for the RT group, the reference formulation of rhGM-CSF was subcutaneously injected on Day 1. After a 7-day washout period, the test formulation of BmrhGM-CSF was orally administered on Day 9.

#### Statistical analysis

The bioavailability was defined in accordance with the *in vivo* bioequivalence guidance criteria established by the US Food and Drug Administration [12]. That states it is the rate and extent to which the active ingredient or active moiety is absorbed from a drug product and becomes available at the site of action of the formulations tested. An analysis of variance (ANOVA) for a 2×2 crossover design in ln-transformed C_max_, AUC_0∼12_ and AUC_0∼∞_ was performed to determine the bioavailability. The 90% CIs for the corresponding differences in C_max_, AUC_0∼12_ and AUC_0∼∞_ were calculated. ANOVA was performed using the F ratio. The probability (*P*) of exceeding the limits of acceptance (80%–125%) was obtained by two 1-sided *t* tests described by Schuirmann [13] . The 2 drug formulations were considered bioequivalent if the geometric mean ratios of the C_max_ and AUC were within the predetermined range of 80% to 125% and if *P* for the 90% CIs was <0.05 [12]. Statistical analysis of the drug product pharmacokinetics was carried out using WinNonlin version 5 (Pharsight Corporation, Mountain View, California).

## Results

### Participant Flow

Originally 22 healthy male volunteers were assessed for eligibility from September, 2005 to January, 2006 in Hangzhou City, Zhejiang Province. Two of them were subsequently excluded because they did not meet the inclusion criteria. The twenty volunteers were randomly divided into the TR and RT groups. One of them withdrew the consent resulting in 19 volunteers being analyzed for the primary outcome (Figure 1).

### Bioavailability

**Figure 2:**
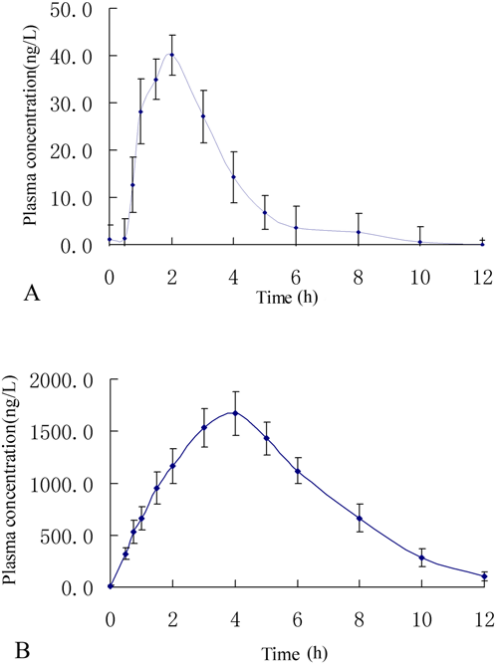
Average hGM-CSF serum concentration versus time in the subjects after either oral administration of BmrhGM-CSF or subcutaneous injection of rhGM-CSF. A: Average hGM-CSF serum concentration versus time in 19 subjects after oral administration of BmrhGM-CSF (8 µg/kg). B: Average hGM-CSF serum concentration versus time in 19 subjects after subcutaneous injection of rhGM-CSF(3.75 µg/kg).

The blood hGM-CSF concentrations of 19 volunteers were determined by ELISA before and after the oral administration of BmrhGM-CSF. Of which, 15 showed significant detectable amount but 4. Figure 2 shows the time course of the average rhGM-CSF serum concentration of 19 subjects who received a single dose of either test BmrhGM-CSF or reference rhGM-CSF. Two hours after the oral administration of BmrhGM-CSF, its absorption peak increased to the amount of 40.1 ng/L. It then returned to the baseline level over the next 10 h.

The values of the hGM-CSF in the sera and time points were fitted using the DASVer 2.0 software. The pharmacokinetics of each of the components was consistent with the two-compartment model. The area under the curve (AUC), C_max_, and T_max_ for the test and reference formulations were calculated using the statistical moment method, and the related pharmacokinetic parameters are shown in Table 1.

**Table 1:** Pharmacokinetic parameters of 19 subjects after the oral administration of 8 µg/kg BmrhGM-CSF or subcutaneous injection of 3.75 µg/kg rhGM-CSF.

Parameters	Unit	BmrhGM-CSF (PO)	rhGM-CSF (SC)
T_max_	h	1.7±0.3	3.9±0.9
C_max_	ng/L	42.6±27.9	1921.5±597.2
AUC_0–12_	ng/h/L	92.12±75.76	10037.01±4127.58

The AUC was calculated using the trapezoid method. The AUC ratio of the test formulation to the reference formulation demonstrated an average bioavailability of the rhGM-CSF in the 19 subjects was 0.61% after oral administration. However, 4 of them showed an undetectable serum concentration of the rhGM-CSF resulting in their AUCs could not be calculated. If these subjects were excluded from the statistic analysis, thus the average bioavailability of the other 15 subjects could reach 1.0%; one of them showed the highest bioavailability of 3.1%.

### MS Tracings

**Figure 3:**
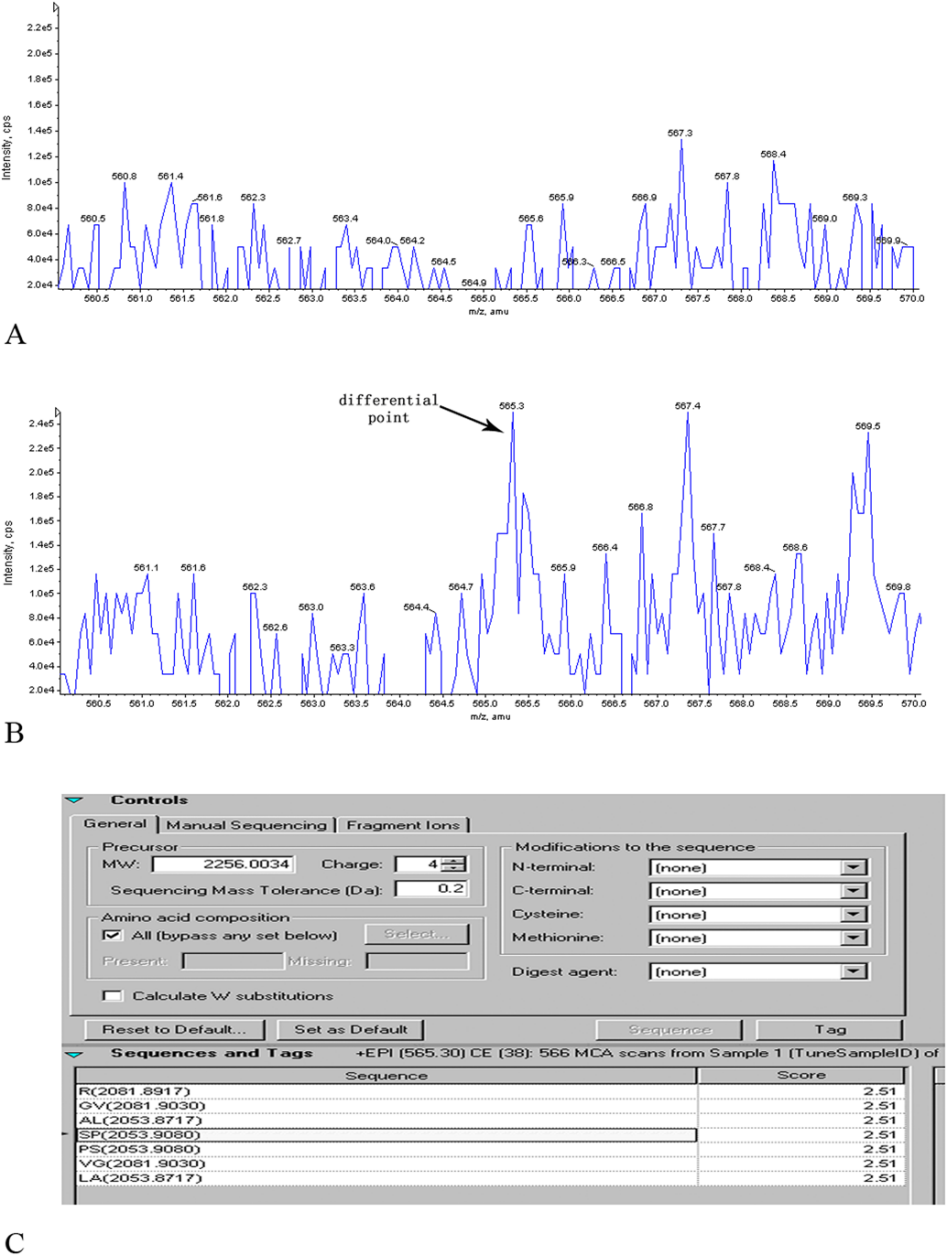
Mass spectrometry profiles of one subject's serum sample after the oral administration of BmrhGM-CSF. A) A mass spectrometry profile of one serum subject's sample after oral administration of BmrhGM-CSF at 0 h. B) A mass spectrometry profile of one subject's serum sample after 3 h of the oral administration of BmrhGM-CSF. The arrow indicates the differential point between A and B. C) An amino acid sequence determined by EPI analysis corresponding to the differential point in one subject's serum sample after 3 h of the oral administration of BmrhGM-CSF in comparison to the hGM-CSF sequence. The SP sequence in the pane is the amino acid sequence corresponding to the differential point.

**Figure 4:**
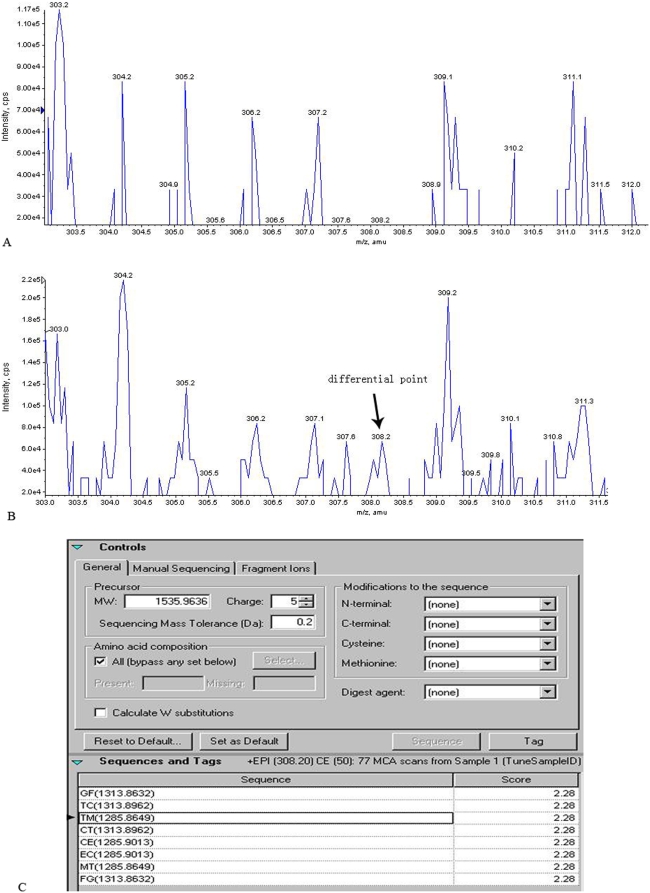
Mass spectrometry profiles of the subject's serum sample after subcutaneous injection of rhGM-CSF. A) A mass spectrometry profile of the serum sample after subcutaneous injection of rhGM-CSF at 0 h. B) A mass spectrometry profile of one subject's serum sample after 3 h of the subcutaneous injection of rhGM-CSF. The arrow indicates the differential point between A and B. C) Amino acid sequence determined by EPI analysis corresponding to the differential point in the subject's serum sample after 3 h of the subcutaneous injection of rhGM-CSF in comparison to the hGM-CSF sequence. The TM sequence in the pane is the amino acid sequence corresponding to the differential point.

Based on the bioavailability data analyses above, the serum samples from three of 15 subjects were used for the MS analyses. Those samples were randomly selected from 15 ELISA detectable subjects that were subjected to either the oral administration of BmrhGM-CSF or the subcutaneous injection of rhGM-CSF. The MS tracings of the 3 subjects as supplementary data were shown in supporting information files ( Table S1, S2, S3 and Figure S1, S2, S3, S4, S5, S6, S7, S8, S9, S10, S11, S12, S13, S14, S15, S16, S17, S18, S19, S20, S21, S22, S23, S24, S25, S26, S27, S28 ). Here, serum MS tracing from one of the 3 volunteers was briefly described as follows:

Using the 0 hour MS data as control, the results of Enhanced Mass Spectrometry (EMS) and EPI analysis suggested that there were differential peaks of the mass-to-electric charge ratio in the serum samples after the oral administration of BmrhGM-CSF or injection of rhGM-CSF (Table 2 and Table 3). Peptide mapping based on the molecular weights demonstrated the presence of hGM-CSF peptide fragments in the differential peaks (Figure 3 and Figure 4). The molecular weights of those different polypeptides were ranged from 2,039 to 7,336Da, which indicated that orally administered BmrhGM-CSF could be absorbed into the blood after penetrated through the intestinal tract.

**Table 2:** EMS and EPI detection results of rhGM-CSF in the serum after either oral administration of BmrhGM-CSF or subcutaneous injection of rhGM-CSF.

MS Sample		Mass-to-electric charge ratio (M/E) determined by EMS for differential peaks of 1h, 2h, 3h, 4h serum samples compared to the 0h serum sample*	EPI analysis in comparison with the hGM-CSF sequence**
PO	1 h	—	—
	2 h	609.4;612.1	—
	3 h	565.3	SP (Figure 3.)
		589.4	—
	4 h	510.8	PN
		589.4; 609.4; 611.3	—
SC	1 h	629.6; 896.5; 879.5; 900.9	—
	2 h	899.3; 919.3; 1284.4; 1359.6	—
	3 h	308.2	TM (Figure 4.)
		363.2	HY
		377.2	QH
		378.1;657.0;898.9	—
		395.3	LTK
		719.6	QT
		996.8; 1027.9;1096.7	—
	4 h	609.4; 765.1; 1475.4	—

* The digital signal is the mass-to-electric charge ratio that corresponds to the peak value in the mass spectrogram. These differential peaks were found in the mass spectrogram of 1h, 2h, 3h, 4h serum samples but 0h sample.

** EPI analysis of the sequences represents partial sequences of the peptide fragment corresponding to differential points, which matched the peptide fragment of hGM-CSF.

**Table 3:** Peptide mass fingerprinting results of rhGM-CSF in the subject serum samples after either oral administration of BmrhGM-CSF or subcutaneous injection of hGM-CSF.

MS Sample	M/E of differential peaks (A)	MW of A	Matched sequence of the peptide fragment of hGM-CSF (B)	Position of B	MW of B	Deviation of MW
PO	565.3	7336.293	TVACSISAPARSPSPSTQPWEHVNAIQEARRLLNLSRDTAAEMNETVEVISEMFDLQEPTCLQTRL	11–76	7336.601	0.308
	510.8	2039.297	HYKQHCPPTPNTSCATQI	100–117	2039.919	0.622
SC	308.2	3070.607	PTPETMCATQIITFESFKENLKDFLLV	107–133	3070.557	0.050
	377.2	4891.481	EVISEMFDLQEPTCLQTRLELYKQHLRGSLTKLKGPLTMMASH	58–100	4891.510	0.029

## Discussion

An oral administration of protein drugs was considered as a focus area in the study of biological pharmaceuticals worldwide. To date, authentic oral administration of protein drugs has not yet been successfully achieved. Most cytokines and proteins including hGM-CSF, interferon and insulin, were found to be effective when delivered by either intramuscular or subcutaneous injection. If these drugs could be effectively administered through the oral administration in clinical settings, the biological pharmaceuticals industry would be revolutionized.

Several challenges could be encountered in the effective oral administration of protein and cytokine drugs which include a poor intrinsic permeability, a hostile proteolytic environment in the gastrointestinal tract, effects of the liver, and short *in vivo* half-lives [14] . Many pharmacologists have been trying to solve these problems. Previous methods for the oral administration of vulnerable pharmacological agents relied on the co-administration of adjuvants (e.g., resorcinol and nonionic surfactants such as polyoxyethylene oleyl ether and n-hexadecyl polyethylene ether) to artificially increase the permeability of the intestina1 walls. Enzymatic inhibitors such as pancreatic trypsin inhibitor and trasylol were also co-administered to inhibit enzymatic degradation [15]. Liposome or microsphere was also used as drug delivery systems for insulin and heparin [16] . Over the past few decades, significant efforts have been made to develop effective oral peptide and protein formulations, and these have met with various degrees of success. Some modified polypeptides or cytokines such as rIL-12, Ile-Pro-[^14^C] Pro, rhG-CSF and insulin were shown to be active after oral administration in animal models [14], [17]–[20]. Several oral preparations of polypeptides and cytokines such as Colostrinin and interferon-alpha have been or still are in a clinical research phase [21]–[24]. However, the bioavailability of protein and cytokine drugs after oral administration in human has rarely been reported.

Foreign genes have been expressed in high level in the silkworm bioreactor. The expressed products possess native-like properties. Additionally, the expressed proteins could be suitably phosphorylated and glycosylated in the bioreactor [25], [26] . Silkworm pupa also harbors an abundance of liposome containing protease inhibitors that protect recombinant proteins from degradation in the gastrointestinal tract [27]–[32]. That allows increased absorption of protein drugs and cytokines, for example, BmrhGM-CSF after the oral administration.

The development of protein drugs that could survive the journey through the gastrointestinal (GI) tract, and then penetrate the bloodstream of the body is a major issue in the pharmaceutical field. Several kinds of protein drugs for oral administration have been genetically engineered. Those include liposome-encapsulated proteins, nanosphere conjugated proteins and fusion proteins which facilitate absorption through the GI tract [14], [17]–[20], [24] . Yet, most of studies are still processing in the stage of animal studies [17]–[20] . It is important to provide experimental proofs at the molecular level to support the theory for the effectiveness of orally administered cytokine drugs or proteins in clinical settings. To achieve this goal, we have carried out a number of preclinical and clinical studies. Here we specifically discuss the clinical data on the bioavailability of BmrhGM-CSF:

In the bioavailability trial, ELISA was used to determine the hGM-CSF concentrations in sera. Using the 19 volunteers, the average hGM-CSF concentration reached its plateau level at 2 h after the oral administration of a single dose of BmrhGM-CSF. The average bioavailability was approximately 1%, and the highest of 3.1%. The blood hGM-CSF concentrations of 19 volunteers were detected by ELISA before and after oral administration of BmrhGM-CSF, but it was not detected in 4 of the 19 ones. This indicated that BmrhGM-CSF could not be absorbed by 4 of 19 subjects. However, BmrhGM-CSF could be absorbed by 79% subjects tested. This might result from individual differences.

The result of MS analysis demonstrated that both small and large hGM-CSF peptide fragments were absorbed into the blood after the oral administration of BmrhGM-CSF, which is in accordance with that of an isotope hybridization test [10].

In this study, we developed a method for the oral administration of protein or cytokine drugs expressed in the silkworm (*B. mori*) bioreactor. The absorption of orally administered rhGM-CSF produced in *B. mori* bioreactor was acceptable, indicating that BmrhGM-CSF may have potential clinical applications. This paper may provide an approach for the oral administration of cytokine and protein drugs.

## Supporting Information

Protocol S1 Bioavailability of orally administered BmrhGM-CSF in healthy volunteers.
(0.08 MB DOC)

Checklist S1 CONSORT Checklist STUDY: Bioavailability of Orally Administered BmrhGM-CSF.
(0.05 MB DOC)

Table S1 Subject NO 1:Table. EMS, EPI and Peptide mass fingerprinting detection results of rhGM-CSF in the plasma after either the oral administration of BmrhGM-CSF or the subcutaneous injection of hGM-CSF.
(0.06 MB DOC)

Table S2 Subject NO 2:Table. EMS, EPI and Peptide mass fingerprinting detection results of rhGM-CSF in the plasma after either the oral administration of BmrhGM-CSF or the subcutaneous injection of hGM-CSF.
(0.06 MB DOC)

Table S3 Subject NO 3:Table. EMS, EPI and Peptide mass fingerprinting detection results of rhGM-CSF in the plasma after either the oral administration of BmrhGM-CSF or the subcutaneous injection of hGM-CSF.
(0.05 MB DOC)

Figure S1 (0.13 MB TIF)

Figure S2 (0.11 MB TIF)

Figure S3 (0.13 MB TIF)

Figure S4 (0.11 MB TIF)

Figure S5 (0.13 MB TIF)

Figure S6 (0.11 MB TIF)

Figure S7 (0.11 MB TIF)

Figure S8 (0.11 MB TIF)

Figure S9 (0.12 MB TIF)

Figure S10 (0.11 MB TIF)

Figure S11 (0.12 MB TIF)

Figure S12 (0.12 MB TIF)

Figure S13 (0.12 MB TIF)

Figure S14 (0.12 MB TIF)

Figure S15 (0.13 MB TIF)

Figure S16 (0.10 MB TIF)

Figure S17 (0.13 MB TIF)

Figure S18 (0.11 MB TIF)

Figure S19 (0.12 MB TIF)

Figure S20 (0.11 MB TIF)

Figure S21 (0.12 MB TIF)

Figure S22 (0.11 MB TIF)

Figure S23 (0.13 MB TIF)

Figure S24 (0.11 MB TIF)

Figure S25 (0.13 MB TIF)

Figure S26 (0.12 MB TIF)

Figure S27 (0.13 MB TIF)

Figure S28 (0.11 MB TIF)
