# Supplementary material for: Bioavailability of Orally Administered rhGM-CSF: A Single-Dose, Randomized, Open-Label, Two-Period Crossover Trial
Source: PLoS One. 2009 May 12;4(5):e5353. doi: 10.1371/journal.pone.0005353 (PMC2677157; doi:10.1371/journal.pone.0005353)
Supplement: Protocol S1 — Bioavailability of orally administered BmrhGM-CSF in healthy volunteers. (0.08 MB DOC) [file pone.0005353.s001.doc]

# **Protocol**

Bioavailability of orally administered bmrhGM-CSF in healthy volunteers

College of Life Sciences, Zhejiang University

2nd Affiliated Hospital of Zhejiang University School of Medicine

Hangzhou，China

Index

Preface [3](#__RefHeading___Toc6131941)

Introduction [3](#__RefHeading___Toc6131942)

Objective 3

Study design 3

Subjects 4

Inclusion criteria 4

Exclusion criteria 4

Supplements 5

Safety of the supplements 5

Restrictions during the study 5

Selection of volunteers 5

Measurements 6

Pre-study measurements: screening 6

In-study measurements 6

Power calculation 6

Compensation for volunteers 7

Advantage for participants of the study 7

Insurance 7

Data collection 7

SAE report 7

References 8

# Preface

This study is performed at 2nd Affiliated Hospital of Zhejiang University School of Medicine， Hangzhou City, China).

The current study is a phase I clinical trial of orally administered BmrhGM-CSF, which is intended to evaluate the bioavailability of orally administered BmrhGM-CSF in healthy volunteers.

# Introduction

The recombinant human granulocyte-macrophage colony-stimulating factor (rhGM-CSF) acts on precursor cell production in the bone marrow. It also stimulates granulocytes, monocytes, and colony formation, and induces hyperplasy of macrophages [1]. This protein is primarily used in the bone marrow transplantation, [tumor chemotherapy](http://dict.cnki.net/dict_result.aspx?r=1&t=tumor+chemotherapy&searchword=肿瘤化疗), and the treatment of aplastic anemia and agranulocytosis related to AIDS [2-6]. The recombinant hGM-CSF expressed by silkworm pupae termed as BmrhGM-CSF, retained a native property. The results of preclinical animal experiments have proven that the oral administration of BmrhGM-CSF could function as an active cytokine [7]. Western blotting analyses have indicated that BmrhGM-CSF could be absorbed into blood through the intestina parva. It was showed that the orally administered BmrhGM-CSF could be absorbed by mice, beagles, and macaques and lead to increased leukocyte counts in these animals. Hematogenesis-inhibited beagles treated with ^60^Co and the orally administered BmrhGM-CSF had white blood cell counts that reached 50% of the baseline value by Day 9 and recovered to the normal levels by Day 12. The oral administration of BmrhGM-CSF to cyclophosphamide-treated macaques increased the leukocyte production in a manner similar to that of leucomax injections. Additionally, the pre-clinical toxicicity tests for BmrhGM-CSF were finished and proven safe. Phase I clinical trials of the orally administered BmrhGM-CSF were approved by the State Food and Drug Administration of P.R.China (authorized document NO. 2003L02511).

# Objectives

The objectives of this study are to 1) evaluate the bioavailability of orally administered BmrhGM-CSF in healthy volunteers, and 2) prove that the orally administered BmrhGM-CSF could be absorbed into blood after passing through the intestinal tract.

# Study design

- Randomized, open-label, two-period crossover study
- A nintervention period of 1 day; a wash-out period of 1 week
- Main outcome: hGM-CSF concentrations in plasma

|  | **Day** | | | |
| --- | --- | --- | --- | --- |
|  | **−6** | **1** | **2-8** | **9** |
| Treatment |  | TR: BmrhGM-CSF (orally administered)  RT:rhGM-CSF (subcutaneously injected) | Wash out | TR: rhGM-CSF (subcutaneously injected)  RT: BmrhGM-CSF (orally administered) |
|  |
| Measurements  Pre-study (screening) | 🡹 |  |  |  |
| Measurements in-study |  | 🡹🡹 |  | 🡹🡹 |

The test formulationwas administered to the TR group on Day 1. After a 7-day washout period, the reference preparation was given on Day 9. The same protocol was performed, vice versa, for the RT group as described above.

# Subjects

Nineteen healthy men aged 18-40 years will participate. We aim at enrolling 22 men for the screening measurement.

## Inclusion criteria

- Apparently healthy
- Age: 18-40 years old
- No liver, kidney, gastrointestinal and metabolic disorders, or neuropsychiatric diseases.
- No medical history, drug allergies history or smoking or alcoholic drinking.
- Did not participate in any drug tests or use any other drugs within the two weeks.
- 18 kg/m^2^ ≤ BMI ≤ 25 kg/m^2^.
- with good communication between researchers, to comply with the requirements of the entire study.
- Signed informed consent and conducted a comprehensive history and physical examination and laboratory tests including blood pressure, heart rate and body temperature measurements, blood examination, urine analysis, blood biochemistry and ECG, etc. Successful applicants have access to the trial.

## Exclusion criteria

- Other clinical trial participants in last 2 months.
- The blood donars during the trial period or within last 1 month.
- No physical examination before the trial.
- Drug allergy or a history of allergic disease.
- History of drug abuse
- Any chronic or acute disease

# Supplements

The following supplements will be given one administration a day in a random order.

- The test formulation of the orally administration of 8 µg/kg with BmrhGM-CSF
- The reference formulation of subcutaneous injection with 3.75 µg/kg of rhGM-CSF

## Safety of supplements

The test formulation of the orally administered BmrhGM-CSF and the reference formulation of the subcutaneously injected rhGM-CSF have been used in previous studies and are safe (8-12).

# Restrictions during the study

#### Diet

During the experiments, the subjects are not allowed to smoke or drink alcohol, caffeine-containing beverages, or fruit juice in order to avoid any possible effect on the absorption and metabolism of the drugs.

Restrictions on the day before the measuring day

- On the day before each measuring day all subjects will receive a standard diet based on their normal energy intake. Breakfast, lunch and diner will be provided by 2nd Affiliated Hospital of Zhejiang University School of Medicine. The diet will consist of normal products, but foods rich in protein and fat will be avoided. Subjects are not allowed to consume any of their own foods during the experiments.
- The evening meal should be ingested prior to 8:00 pm.
- All of subjects are not allowed to eat or drink after 10:00 pm.

Restrictions on the testing day

Subjects fasted overnight for at least 10 h. At 8:00 am on the following day, subjects who had abstained from drinking water 2 h before the oral administration of BmrhGM-CSF are given the drug with 200 ml warm boiled water. After 4 h, all of the volunteers eat the same low-fat meal. Subjects are not allowed to smoke, eat or drink (except water) until the blood sample collection has completed.

Other

Physical activity and dietary habit should remain constant during the study.

# Selection of volunteers

Subjects will be orally informed about the study via an information meeting and in writing of an information brochure. Subjects will sign an informed consent before the beginning of the study.

Recruitment procedure:

1. Recruit subjects by mailing information through local municipalities
   - Letter
   - Flyer with a short description of the study
   - Application card for information
2. Mailing of information to subjects who returned the application card
   - Information brochure
   - Informed consent
   - Medical questionnaire
3. Invitation of subjects for the information meeting, based on return of informed consent and eligibility criteria from a medical questionnaire
4. Information meeting
5. Screening
   - height/weight measurement
   - blood sample
   - urine sample
6. Nineteen eligible subjects, based on in/exclusion criteria are included in the study

# Measurements

## Pre-study measurements: screening

- weight
- height
- blood pressure
- blood measurements:
- hematology
- blood biochemical analyses (kidney and liver functions, and levels of blood sugar )
- electrocardiography
- urine analysis
- medical questionnaire

## In-study measurements

Measurements after an overnight fast:

- hGM-CSF concentration in plasma after drug administration at 0, 0.5, 0.75, 1.0, 1.5, 2.0, 3.0, 4.0, 5.0, 6.0, 8.0, 10.0, and 12.0 h

# Power calculation

The power analysis is done based on changes in hGM-CSF concentrations in plasma after drug administrations at 0, 0.5, 0.75, 1.0, 1.5, 2.0, 3.0, 4.0, 5.0, 6.0, 8.0, 10.0, and 12.0 h.

# Compensation for volunteers

Volunteers who complete the study will receive RMB 800 Yuan.

If a volunteer decides to discontinue the study, the volunteer will be compensated proportionally. Travel costs will be reimbursed.

# Advantage for participants of the study

This study has no direct advantage for participants of this study. From a public health point of view the results of this study can contribute in the future to the assessment as to whether BmrhGMCSF is to increase leukocyte counts in tumor patients received chemotherapy or radiotherapy for use by oral administration.

# Insurance

2nd Affiliated Hospital of Zhejiang University School of Medicine has an effective insurance policy (normal risk) for each volunteer in the study. Volunteers will be informed about the insurance.

# Data collection

All the data are collected at 2nd Affiliated Hospital of Zhejiang University School of Medicine.

# SAE REPORT

# Any serious adverse events during the trial should be reported to pharmaceutical supervision and management departments (SFDA) and the Medical Ethics Committee within 24 hours.

# References

1. Gasson J.C (1991) Molecular physiology of granulocyte-macrophage colony-stimulating factor. Blood 77, 1131–1145.

2. Vadhan-Raj S, Keating M, LeMaistre, A., Hittelman WN, McCredie K, et al. (1987) Effects of recombinant human granulocyte-macrophage colony-stimulating factor in patients with myelodysplastic syndromes. N. Engl. J. Med. 317, 1545–1552.

3. Antman KS, Griffin JD, Elias A, Socinski MA, Ryan L, et al. (1988) Effect of recombinant human granulocyte-macrophage colony-stimulating factor on chemotherapy-induced myelosuppression. N. Engl. J. Med. 319, 593–598.

4. Hord JD, Gay JC, Whitlock JA, Janco RL, Edwards JR, et al. (1995) Long-term granulocyte-macrophage colony-stimulating factor and immunosuppression in the treatment of acquired severe aplastic anemia. J. Pediatr. Hematol. Oncol. 17, 140–144.

5. Badaro R, Nascimento C, Carvalho JS, Badaro F, Russo D, et al. (1994) Recombinant human granulocyte-macrophage colony-stimulating factor reverses neutropenia and reduces secondary infections in visceral leishmaniasis. J. Infect. Dis. 170, 413–418.

6. Hardy D, Spector S, Polsky B, Crumpacker C, Van der Horst C, et al. (1994) Combination of ganciclovir and granulocyte-macrophage colony-stimulating factor in the treatment of cytomegalovirus retinitis in AIDS patients. Eur. J. Clin. Microbiol. Infect. Dis. 13 (Suppl. 2), S34–S40.

7. Zhang YZ, Chen J, Lv ZB, Nie ZM, Zhang XY,et al. (2006) Can 29 kDa rhGM-CSF expressed by silkworm pupae bioreactor bring into effect as active cytokine through orally administration? Eur. J. Pharmaceut. Sci. 28, 212–223.

8. Thompson JA, Lee DJ, Kidd P, et al. (1989) Subcutaneous granulocyte-macrophage colony-stimulating factor in patients with myelodysplastic syndrome: toxicity, pharmacokinetics, and hematological effects. J Clin Oncol.7(5):629-637.

9. Cebon JS, Bury RW, Lieschke GJ and Morstyn G. (1990) The effects of dose and route of administration on the pharmacokinetics of granulocyte-macrophage colony-stimulating factor. Eur J Cancer.26(10):1064-1069.

10. Cebon J, Dempsey P, Fox R, et al. (1988) Pharmacokinetics of human granulocyte-macrophage colony-stimulating factor using a sensitive immunoassay. Blood.72(4):1340-1347.

11. Aweeka F,Kwong M, Mak M, et al. (1996) Pharmacokinetics of recombinant human granulocyte-macrophage colony-stimulating factor: the effects of zidovudine. J Clin Pharmacol.36:1107-1113.

12. Burchiel SW, Oette D, Day P and Stoli RE. (1994) Analysis of radiolabeled cho cell-derived rhuGM-CSF pharmacokinetics and biodistribution in rhesus monkeys following intravenous and subcutaneous injection. Int J Immunopharmacol.16:75-90.
