# Supplementary material for: Bioavailability of Orally Administered rhGM-CSF: A Single-Dose, Randomized, Open-Label, Two-Period Crossover Trial
Source: PLoS One. 2009 May 12;4(5):e5353. doi: 10.1371/journal.pone.0005353 (PMC2677157; doi:10.1371/journal.pone.0005353)
Supplement: Table S1 — Subject NO 1:Table. EMS, EPI and Peptide mass fingerprinting detection results of rhGM-CSF in the plasma after either the oral administration of BmrhGM-CSF or the subcutaneous injection of hGM-CSF. (0.06 MB DOC) [file pone.0005353.s003.doc]

**NO 1-Table 1.EMS and EPI detection results of rhGM-CSF in the plasma after either the oral administration of BmrhGM-CSF or the subcutaneous injection of hGM-CSF.**

| MS  Sample | | Mass-to-electric charge ratio (M/E) determined by EMS for differential peaks of 1h, 2h, 3h, 4h plasma samples compared to the 0h plasma sample* | EPI analysis in comparison with the hGM-CSF sequence** |
| --- | --- | --- | --- |
|  |  |  |  |
| PO | 1 h | — | — |
|  | 2 h | 609.4;612.1 | — |
|  | 3 h | 565.3 | SP (Figure S1,2) |
|  |  | 589.4 | — |
|  | 4 h | 510.8 | PN (Figure S3,4) |
|  |  | 589.4; 609.4; 611.3 | — |
| SC | 1 h | 629.6; 896.5; 879.5; 900.9 | — |
|  | 2 h | 899.3; 919.3; 1284.4; 1359.6 | — |
|  | 3 h | 308.2 | TM (Figure S5,6) |
|  |  | 363.2 | HY (Figure S7,8) |
|  |  | 377.2 | QH(Figure S9,10) |
|  |  | 378.1;657.0;898.9 | — |
|  |  | 395.3 | LTK(Figure S11,12) |
|  |  | 719.6 | QT(Figure S13,14) |
|  |  | 996.8; 1027.9;1096.7 | — |
|  | 4 h | 609.4; 765.1; 1475.4 | — |

| MS  Sample | M/E of differential  peaks (A) | MW of A | Matched sequence of the peptide fragment of hGM-CSF (B) | Position  of B | MW of B | Deviation of MW |
| --- | --- | --- | --- | --- | --- | --- |
| PO | 565.3 | 7336.293 | tvacsisaparspspstqpwehvnaiqearrllnlsrdtaaemnetvevisemfdlqeptclqtrl | 11--76 | 7336.601 | 0.308 |
|  | 510.8 | 2039.297 | hykqhcpptpNtscatqi | 100--117 | 2039.919 | 0.622 |
| SC | 308.2 | 3070.607 | PTPETMCATQIITFESFKENLKDFLLV | 107--133 | 3070.557 | 0.050 |
|  | 377.2 | 4891.481 | EVISEMFDLQEPTCLQTRLELYKQHLRGSLTKLKGPLTMMASH | 58--100 | 4891.510 | 0.029 |

*The digital signal is the mass-to-electric charge ratio that corresponds to the peak value in the [mass spectrogram](http://dict.cnki.net/dict_result.aspx?r=1&t=mass+spectrogram&searchword=质谱图).These differential peaks were found in the mass spectrogram of 1, 2, 3, 4h plasma samples but 0h plasma sample. ** By EPI analysis, the sequences represent partial sequences of the peptide fragment corresponding to differential points, which matched the peptide fragment of hGM-CSF.

**NO 1-Table 2.**[**Peptide mass fingerprinting**](http://dict.cnki.net/dict_result.aspx?r=1&t=peptide+mass+fingerprinting&searchword=肽质量指纹分析) **detection results of rhGM-CSF in the plasma after either the oral administration of BmrhGM-CSF or the subcutaneous injection of hGM-CSF.**
