# Supplementary material for: Bioavailability of Orally Administered rhGM-CSF: A Single-Dose, Randomized, Open-Label, Two-Period Crossover Trial
Source: PLoS One. 2009 May 12;4(5):e5353. doi: 10.1371/journal.pone.0005353 (PMC2677157; doi:10.1371/journal.pone.0005353)
Supplement: Table S2 — Subject NO 2:Table. EMS, EPI and Peptide mass fingerprinting detection results of rhGM-CSF in the plasma after either the oral administration of BmrhGM-CSF or the subcutaneous injection of hGM-CSF. (0.06 MB DOC) [file pone.0005353.s004.doc]

**NO-2-Table1.EMS and EPI detection results of rhGM-CSF in the plasma after either the oral administration of BmrhGM-CSF or the subcutaneous injection of hGM-CSF.**

| MS  Sample | | Mass-to-electric charge ratio (M/E) determined by EMS for differential peaks of 1h, 2h, 3h, 4h plasma samples compared to the 0h plasma sample* | EPI analysis in comparison with the hGM-CSF sequence** |
| --- | --- | --- | --- |
|  |  |  |  |
| PO | 1 h | — | — |
|  | 2 h | 616.2 | — |
|  | 3 h | 460.7 | — |
|  |  | 491.0 | GP (Figure S15，16) |
|  |  | 1299.7 | EM(Figure S17，18) |
|  | 4 h | 616.3;1309.7 | — |
| SC | 1 h | 1509.1 | — |
|  | 2 h | 586.4;1309.7; 1439.2;1509.2 | — |
|  | 3 h | 587.7 | SF (Figure S19，20) |
|  |  | 708.7 | AP (Figure S21，22) |
|  |  | 718.4;954.1;1027.0; | — |
|  | 4h | 384.1;587.0;713.5; 715.7;719.9; 1409.3 | — |

*The digital signal is the mass-to-electric charge ratio that corresponds to the peak value in the [mass spectrogram](http://dict.cnki.net/dict_result.aspx?r=1&t=mass+spectrogram&searchword=质谱图).These differential peaks were found in the mass spectrogram of 1, 2, 3, 4h plasma samples but 0h plasma sample.

** By EPI analysis, the sequences represent partial sequences of the peptide fragment corresponding to differential points, which matched the peptide fragment of hGM-CSF.

**NO-2-Table2.**[**Peptide mass fingerprinting**](http://dict.cnki.net/dict_result.aspx?r=1&t=peptide+mass+fingerprinting&searchword=肽质量指纹分析) **detection results of rhGM-CSF in the plasma after either the oral administration of BmrhGM-CSF or the subcutaneous injection of hGM-CSF.**

| MS  Sample | M/E of differential  peaks (A) | MW of A | Matched sequence of the peptide fragment of hGM-CSF (B) | Position  of B | MW of B | Deviation of MW |
| --- | --- | --- | --- | --- | --- | --- |
| PO | 491.0 | 4410.618 | qsllllgpvacsisaparspspstqpwehvnaiqearrlln | 4-44 | 4410.323 | 0.295 |
|  | 1309.7 | 6543.333 | TQPWEHVNAIQEARRLLNLSRDTAAEMNETVEVISEMFDLQEPTCLQTRLELYKQG | 27-82 | 6543.226 | 0.107 |
| SC | 587.0 | 7032.658 | ARRLLNLSRDTAAEMNETVEVISEMFDLQEPTCLQTRLELYKQGLRGSLTKLKGPLTMMASH | 39-100 | 7032.612 | 0.046 |
|  | 954.1 | 2859.494 | TQPWEHVNAIQEARRLLNLSRDTAAEMNETVEVI | 105-138 | 2859.476 | 0.018 |
|  | 713.5 | 3561.742 | MWLQSLLLLGTVACSISAPARSPSPSTQPWEHV | 1-33 | 3561.811 | 0.069 |
