# Supplementary material for: Bioavailability of Orally Administered rhGM-CSF: A Single-Dose, Randomized, Open-Label, Two-Period Crossover Trial
Source: PLoS One. 2009 May 12;4(5):e5353. doi: 10.1371/journal.pone.0005353 (PMC2677157; doi:10.1371/journal.pone.0005353)
Supplement: Table S3 — Subject NO 3:Table. EMS, EPI and Peptide mass fingerprinting detection results of rhGM-CSF in the plasma after either the oral administration of BmrhGM-CSF or the subcutaneous injection of hGM-CSF. (0.05 MB DOC) [file pone.0005353.s005.doc]

**NO-3-Table1 . EMS, EPI and** [**Peptide mass fingerprinting**](http://dict.cnki.net/dict_result.aspx?r=1&t=peptide+mass+fingerprinting&searchword=肽质量指纹分析) **detection results of rhGM-CSF in the plasma after either the oral administration of BmrhGM-CSF or the subcutaneous injection of hGM-CSF.**

| MS  Sample | | Mass-to-electric charge ratio (M/E) determined by EMS for differential peaks of 1h, 2h, 3h, 4h plasma samples compared to the 0h plasma sample* | EPI analysis in comparison with the hGM-CSF sequence** |
| --- | --- | --- | --- |
|  |  |  |  |
| PO | 1 h | — | — |
|  | 2 h | 1637.9 | — |
|  | 3 h | 1632.7 | SF (Figure S23,24) |
|  |  | 1247.0 | — |
|  | 4 h | 965.9;1241.2 | — |
| SC | 1 h | 722.3; 947.6;1681.6 | — |
|  | 2 h | 722.3;867.2;946.2;1081.6 | — |
|  | 3 h | 713.9; | MF(Figure S25,26) |
|  |  | 920.2 | MA(Figure S27,28) |
|  |  | 841.4;1270.0;1500.3 | — |
|  | 4h | 866.9;946.2;957.6; | — |

*The digital signal is the mass-to-electric charge ratio that corresponds to the peak value in the [mass spectrogram](http://dict.cnki.net/dict_result.aspx?r=1&t=mass+spectrogram&searchword=质谱图).These differential peaks were found in the mass spectrogram of 1, 2, 3, 4h plasma samples but 0h plasma sample.

** By EPI analysis, the sequences represent partial sequences of the peptide fragment corresponding to differential points, which matched the peptide fragment of hGM-CSF.

**NO-3-Table2.**[**Peptide mass fingerprinting**](http://dict.cnki.net/dict_result.aspx?r=1&t=peptide+mass+fingerprinting&searchword=肽质量指纹分析) **detection results of rhGM-CSF in the plasma after either the oral administration of BmrhGM-CSF or the subcutaneous injection of hGM-CSF.**

| MS  Sample | M/E of differential  peaks (A) | MW of A | Matched sequence of the peptide fragment of hGM-CSF (B) | Position  of B | MW of B | Deviation of MW |
| --- | --- | --- | --- | --- | --- | --- |
| PO | 1632.7 | 6526.331 | rgsltklkgpltmmashykqhcpptpetscatqiitfesfkenlkdfllvipfdcwe | 84-140 | 6526.233 | 0.098 |
| SC | 1081.6 | 10805.962 | LGTVACSISAPARSPSPSTQPWEHVNAIQEARRLLNLSRDTAAEMNETVEVISEMFDLQEPTCLQTRLELYKQGLRGSLTKLKGPLTMMASHYKQHC | 9-105 | 10806.416 | 0.454 |
|  | 1500.3 | 8996.261 | PSPSTQPWEHVNAIQEARRLLNLSRDTAAEMNETVEVISEMFDLQEPTCLQTRLELYKQGLRGSLTKL KGPLTMMASHY | 23-101 | 8996.518 | 0.257 |
