# Supplementary figures and images for: Bioavailability of Orally Administered rhGM-CSF: A Single-Dose, Randomized, Open-Label, Two-Period Crossover Trial
Source: PLoS One. 2009 May 12;4(5):e5353. doi: 10.1371/journal.pone.0005353 (PMC2677157; doi:10.1371/journal.pone.0005353)

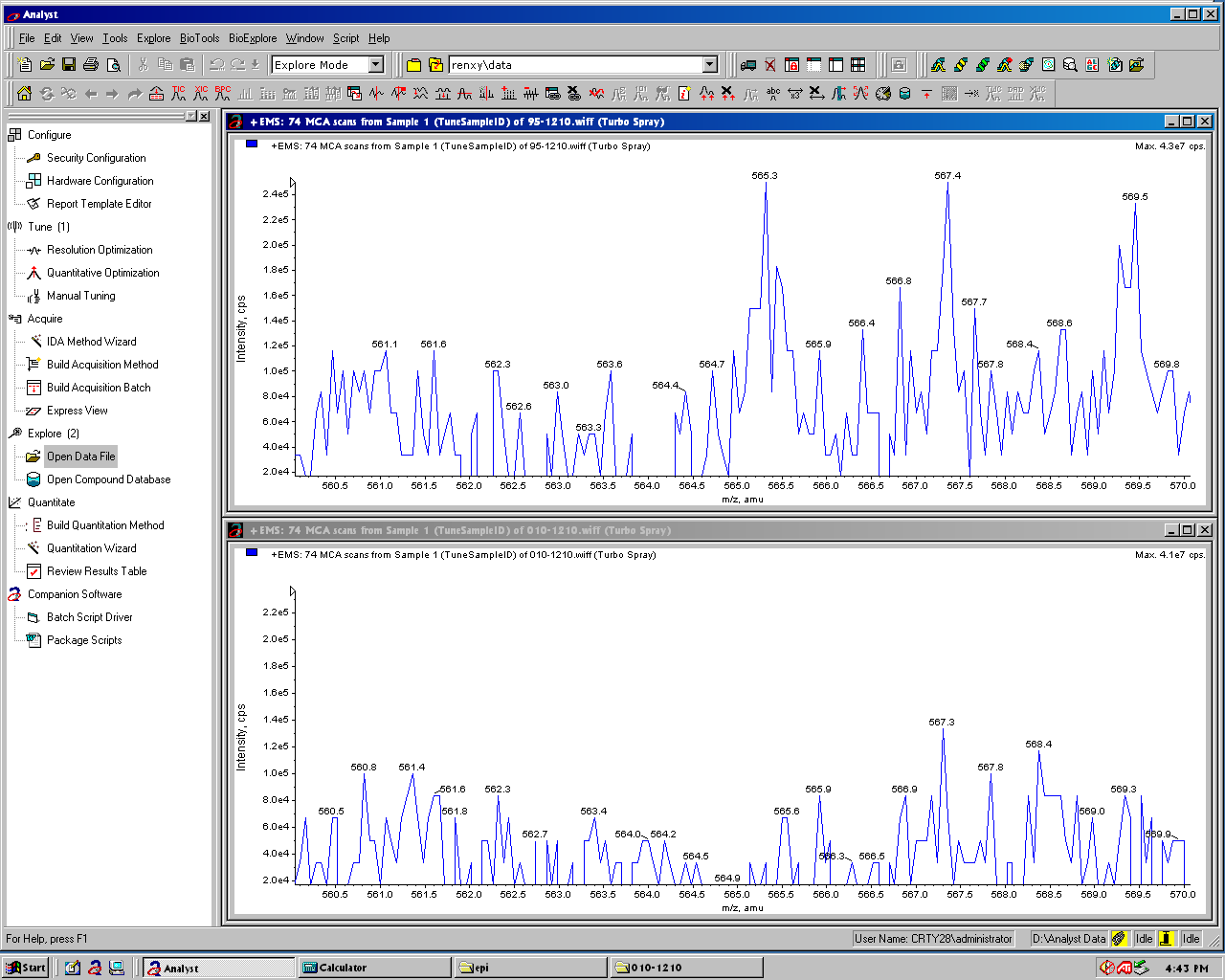

Supplement: Figure S1 — (0.13 MB TIF) [file pone.0005353.s006.tif]

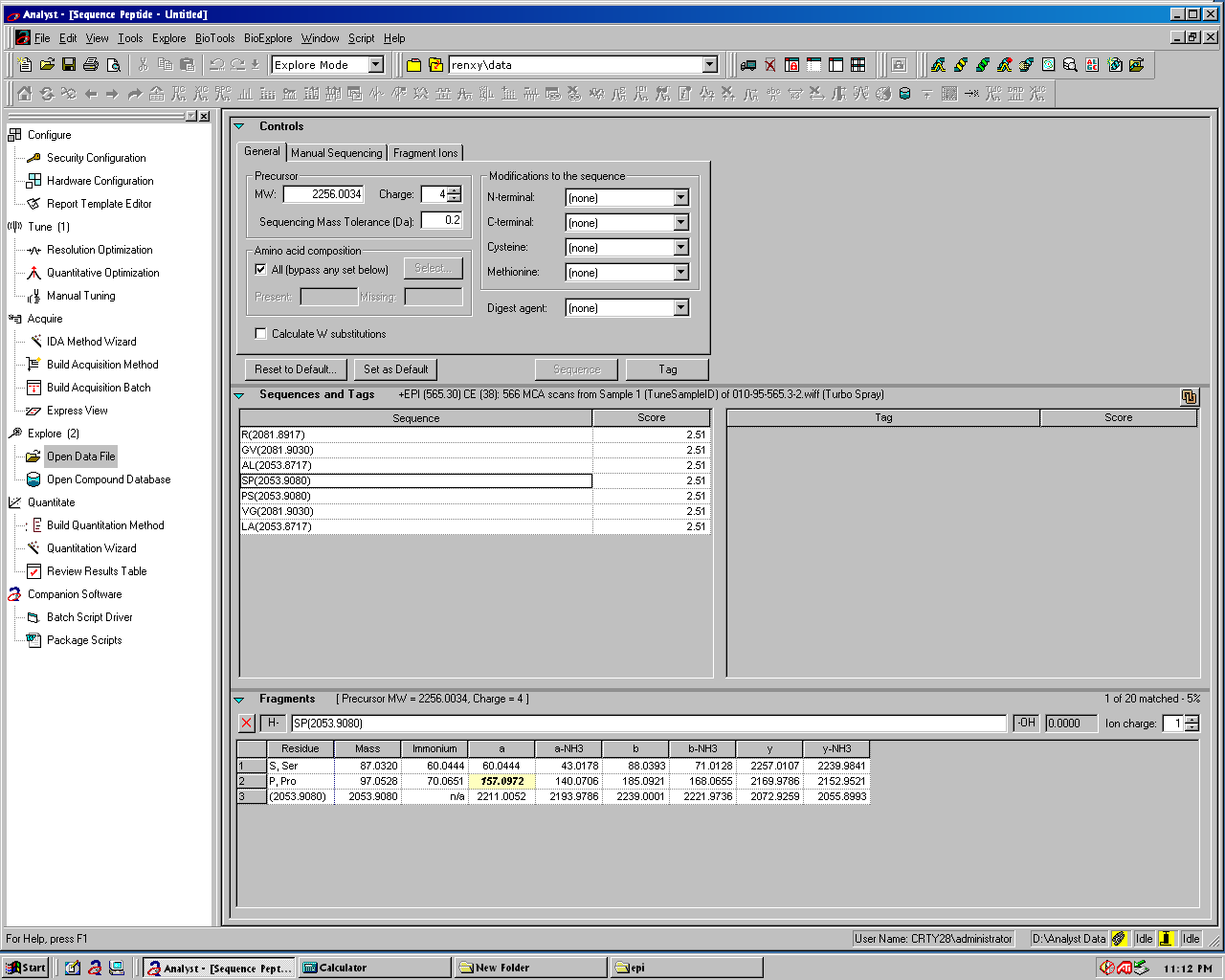

Supplement: Figure S2 — (0.11 MB TIF) [file pone.0005353.s007.tif]

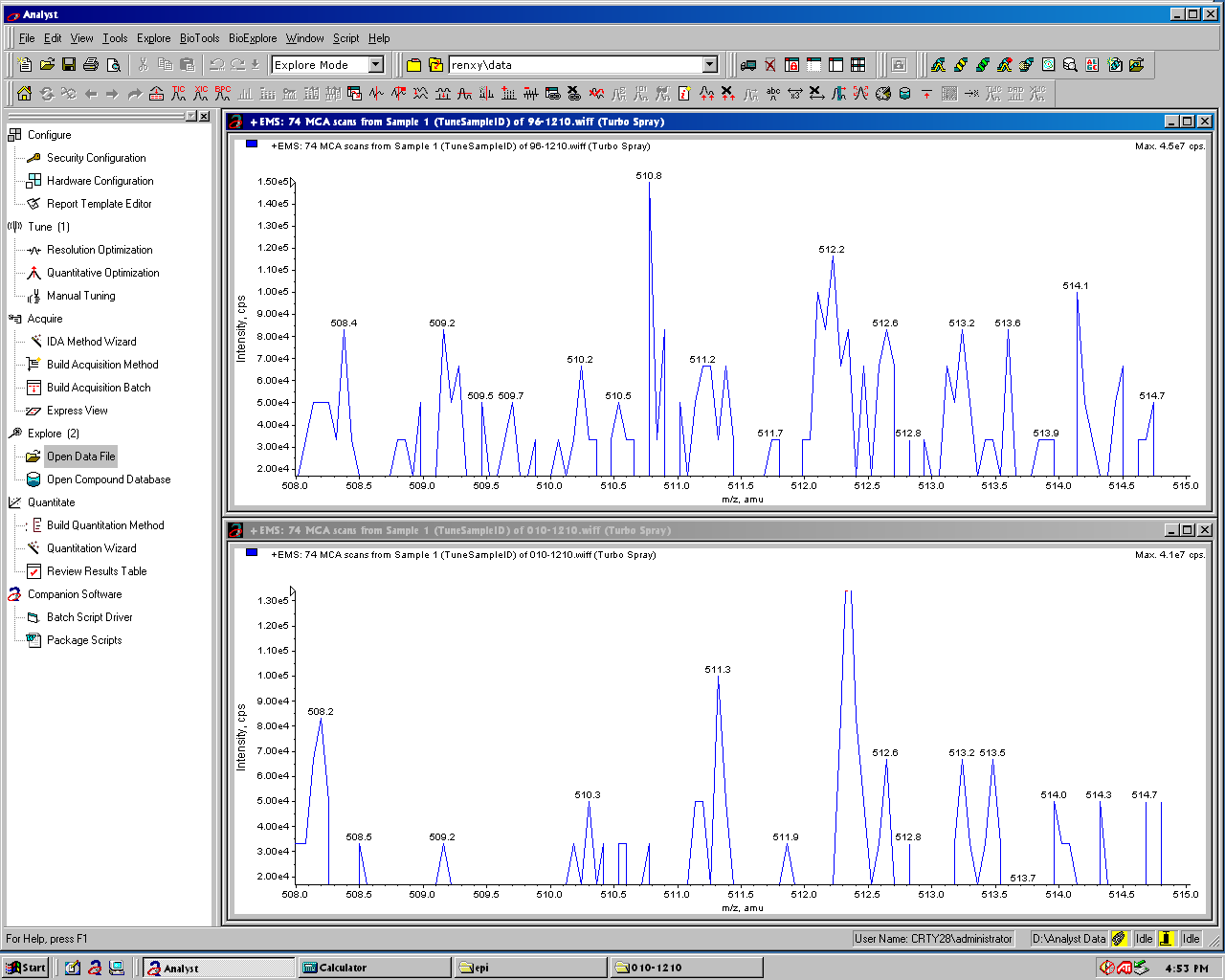

Supplement: Figure S3 — (0.13 MB TIF) [file pone.0005353.s008.tif]

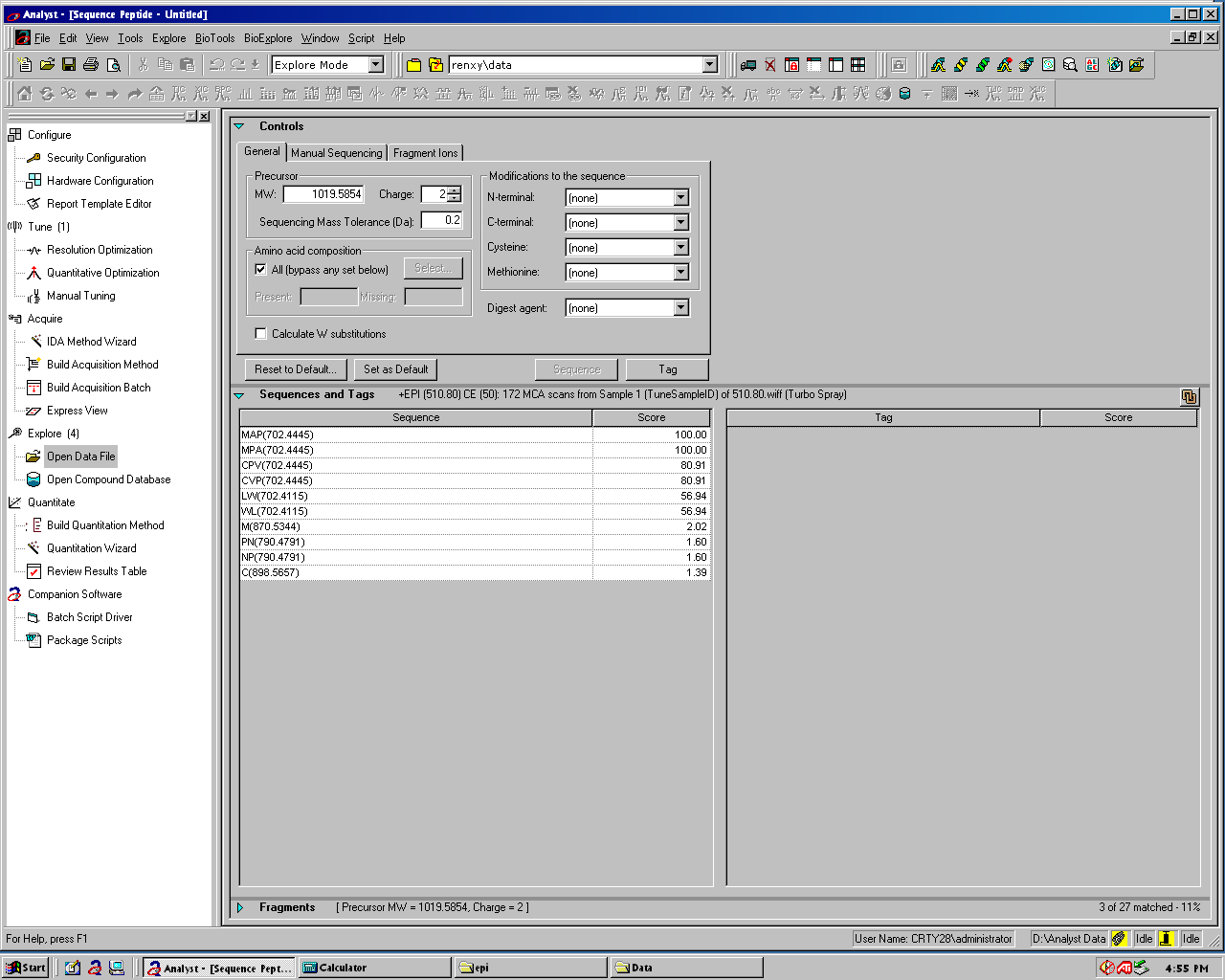

Supplement: Figure S4 — (0.11 MB TIF) [file pone.0005353.s009.tif]

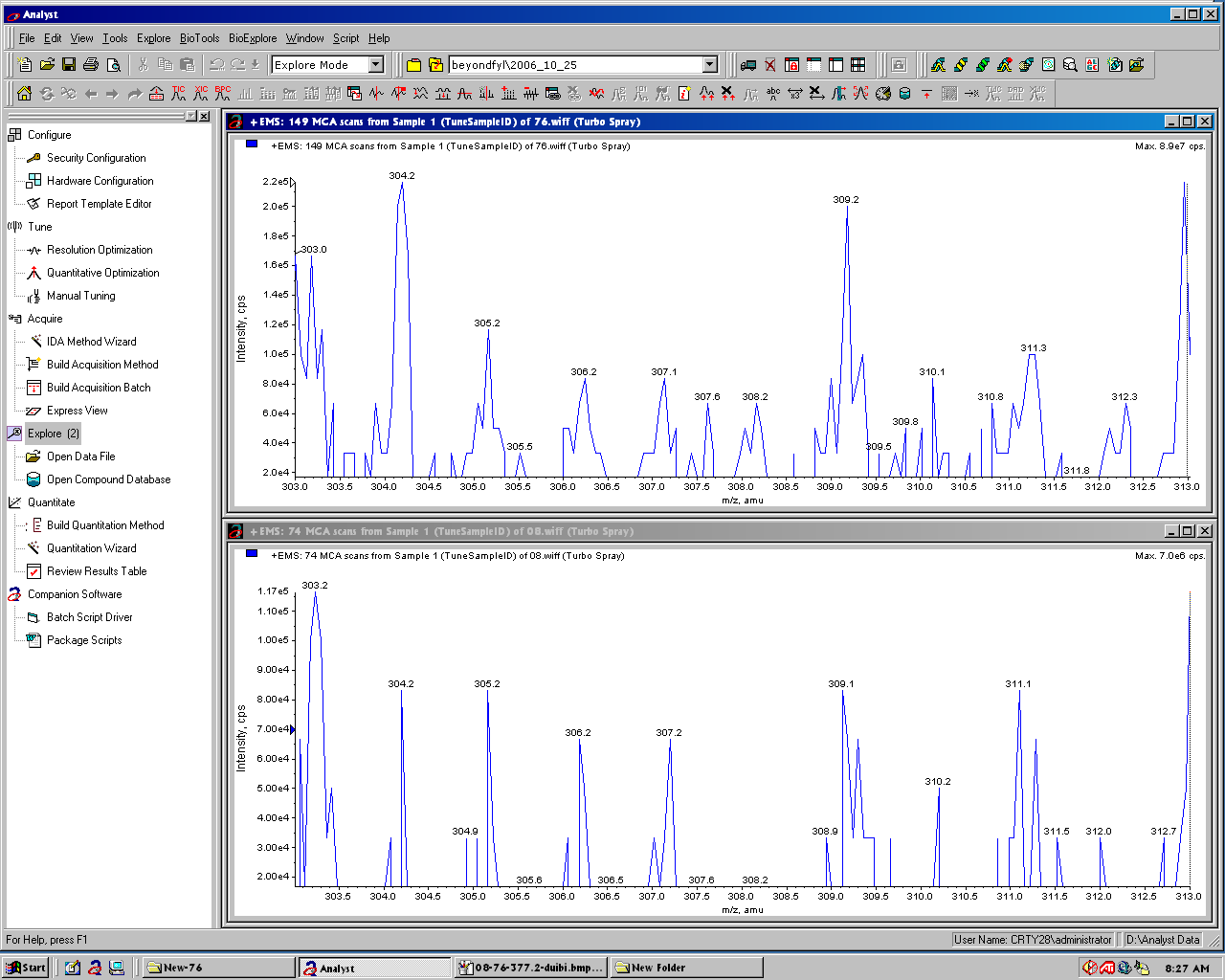

Supplement: Figure S5 — (0.13 MB TIF) [file pone.0005353.s010.tif]

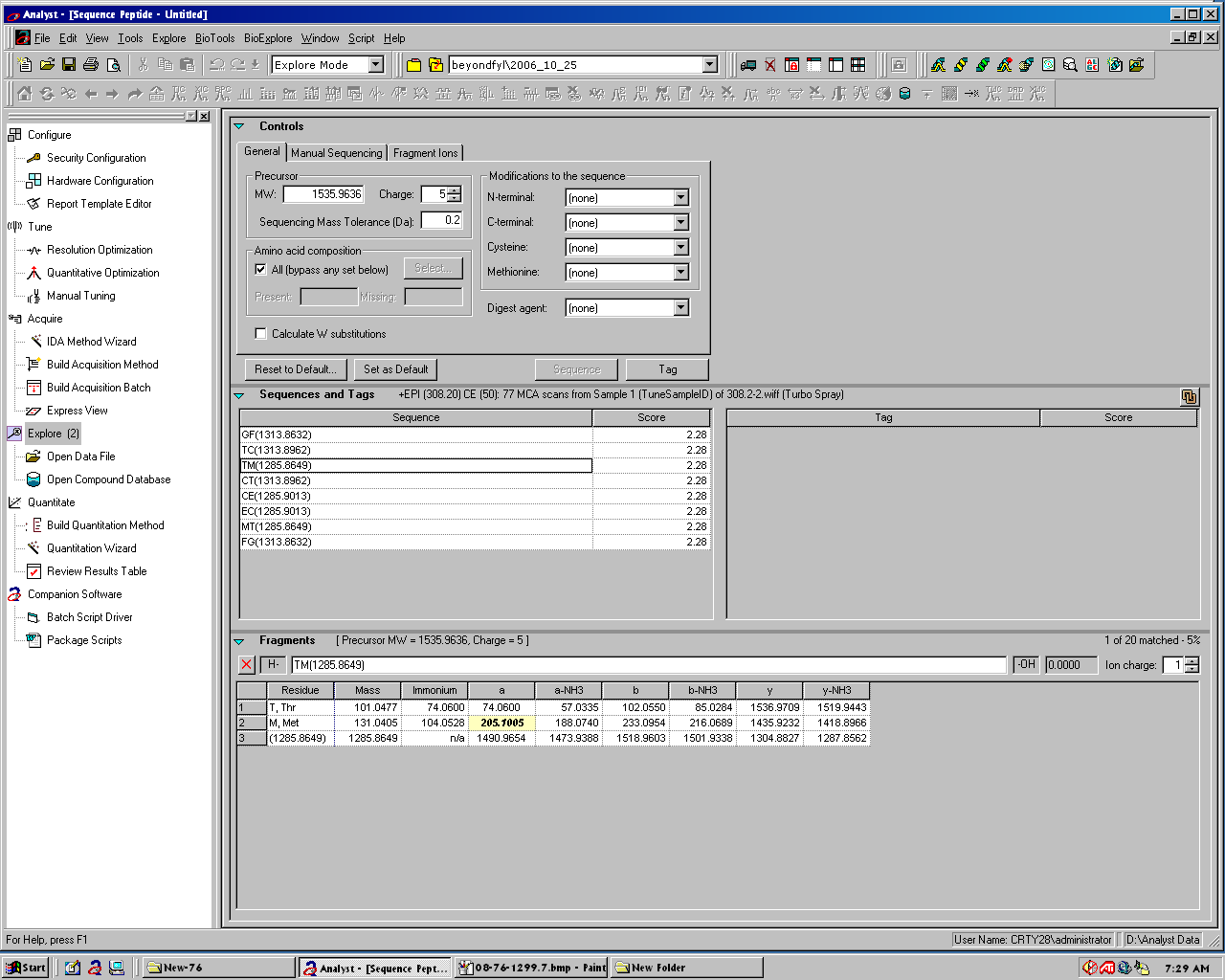

Supplement: Figure S6 — (0.11 MB TIF) [file pone.0005353.s011.tif]

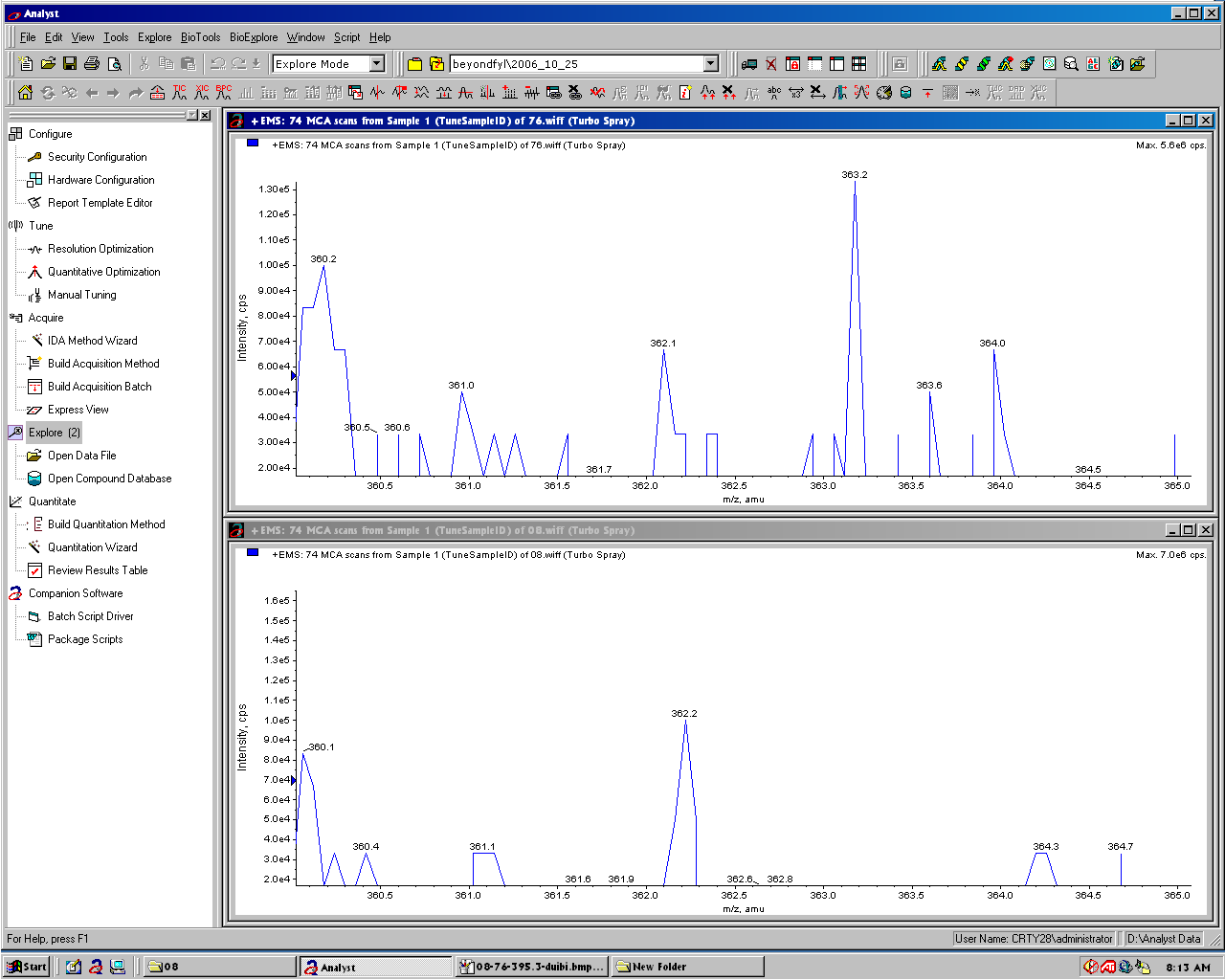

Supplement: Figure S7 — (0.11 MB TIF) [file pone.0005353.s012.tif]

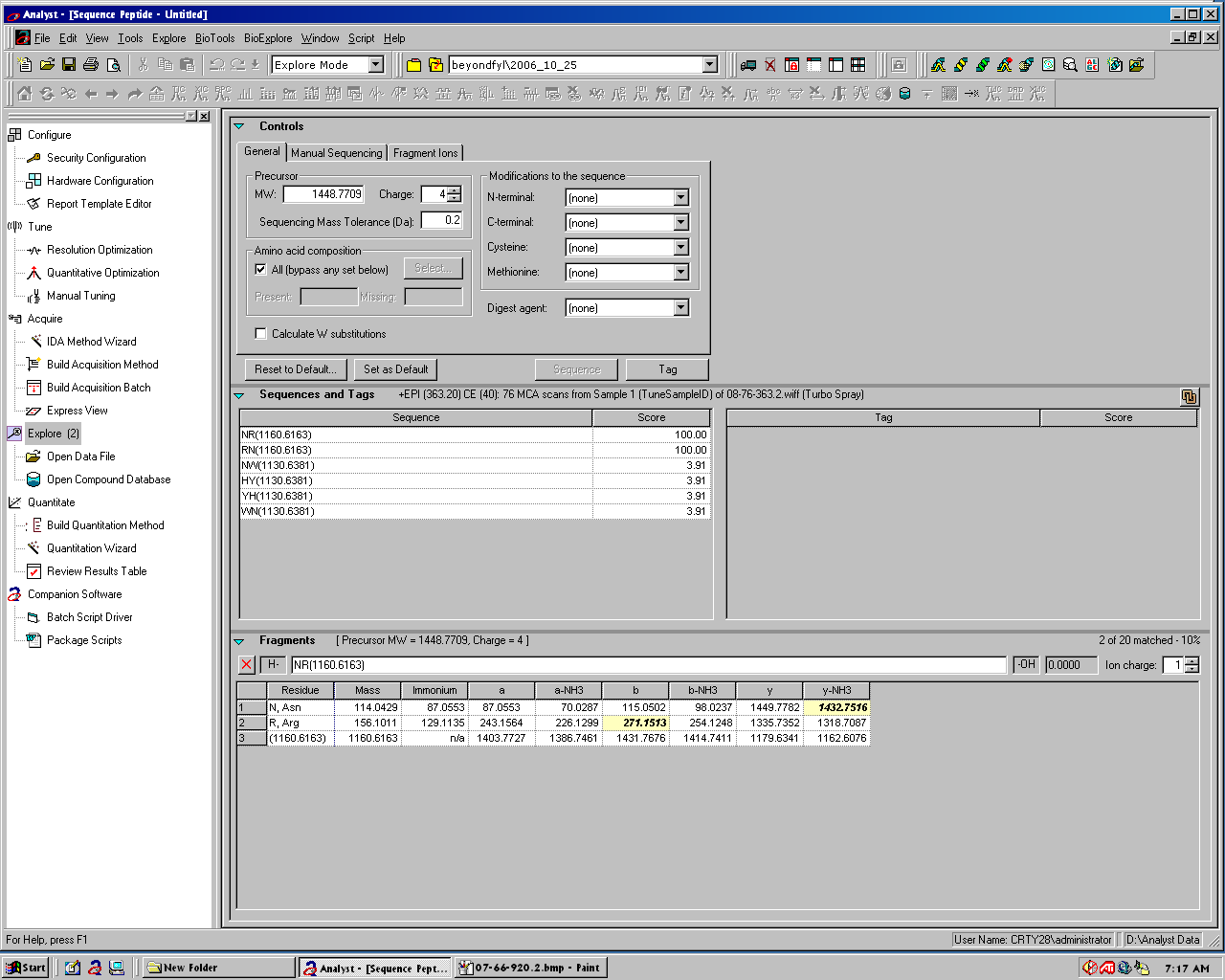

Supplement: Figure S8 — (0.11 MB TIF) [file pone.0005353.s013.tif]

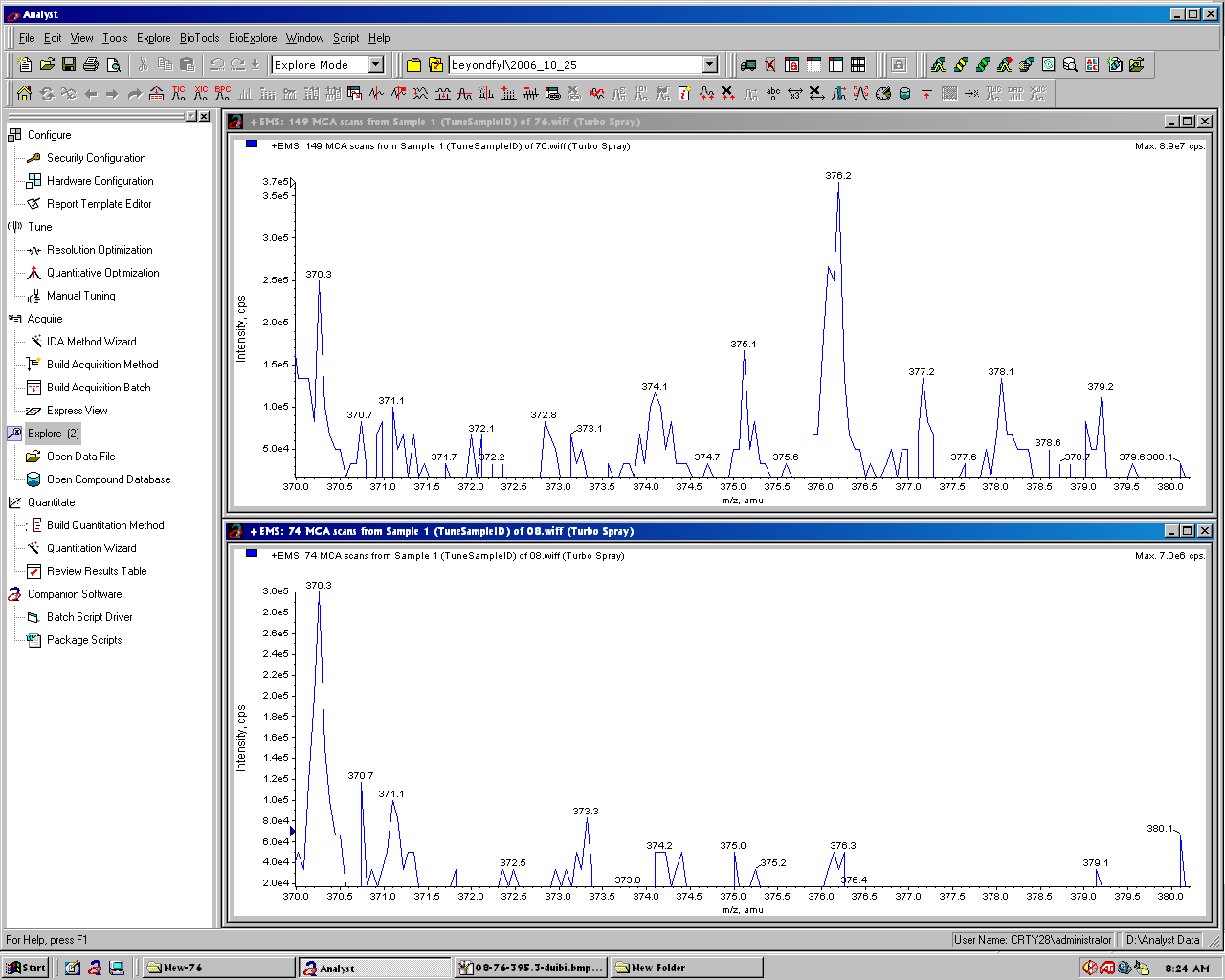

Supplement: Figure S9 — (0.12 MB TIF) [file pone.0005353.s014.tif]

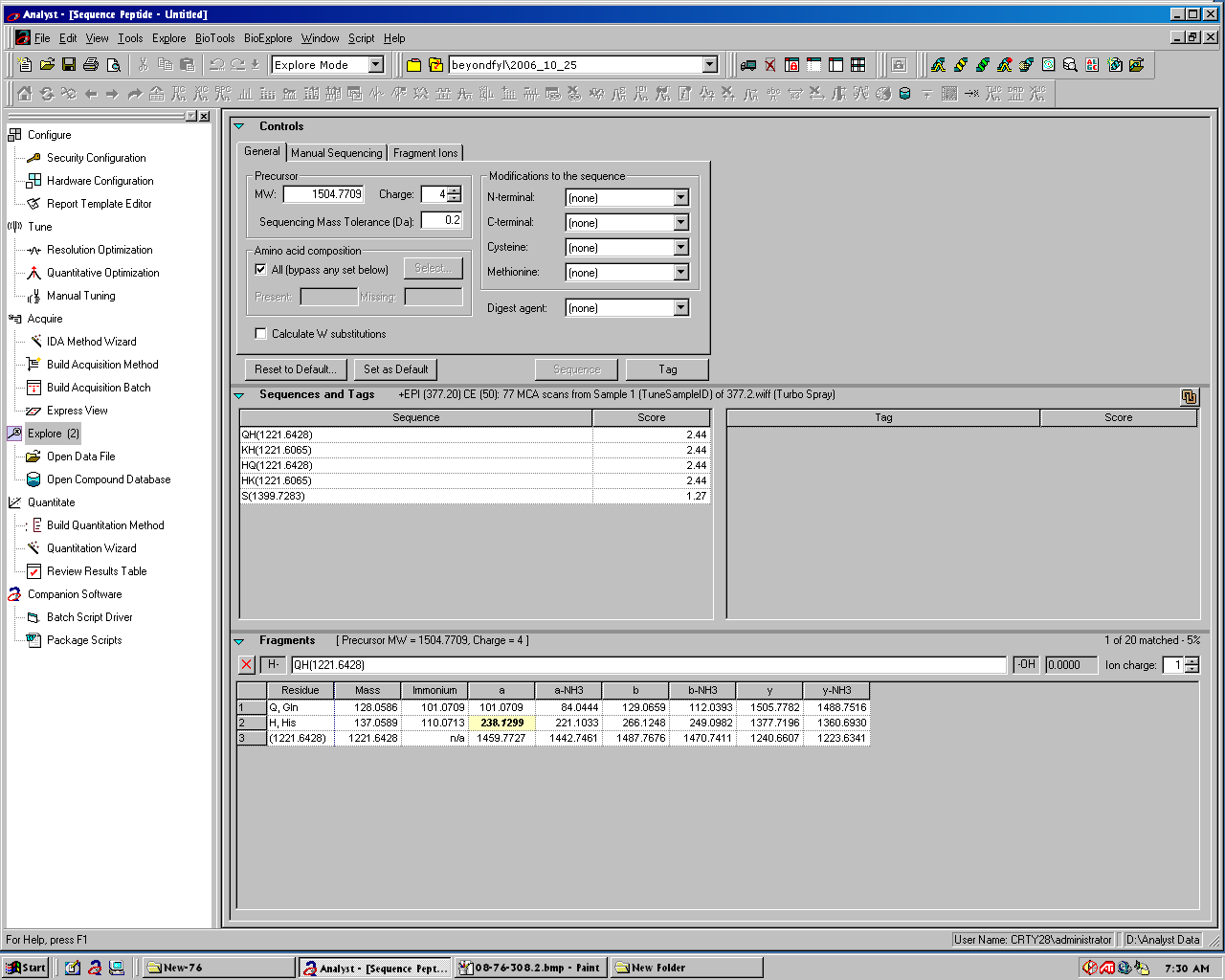

Supplement: Figure S10 — (0.11 MB TIF) [file pone.0005353.s015.tif]

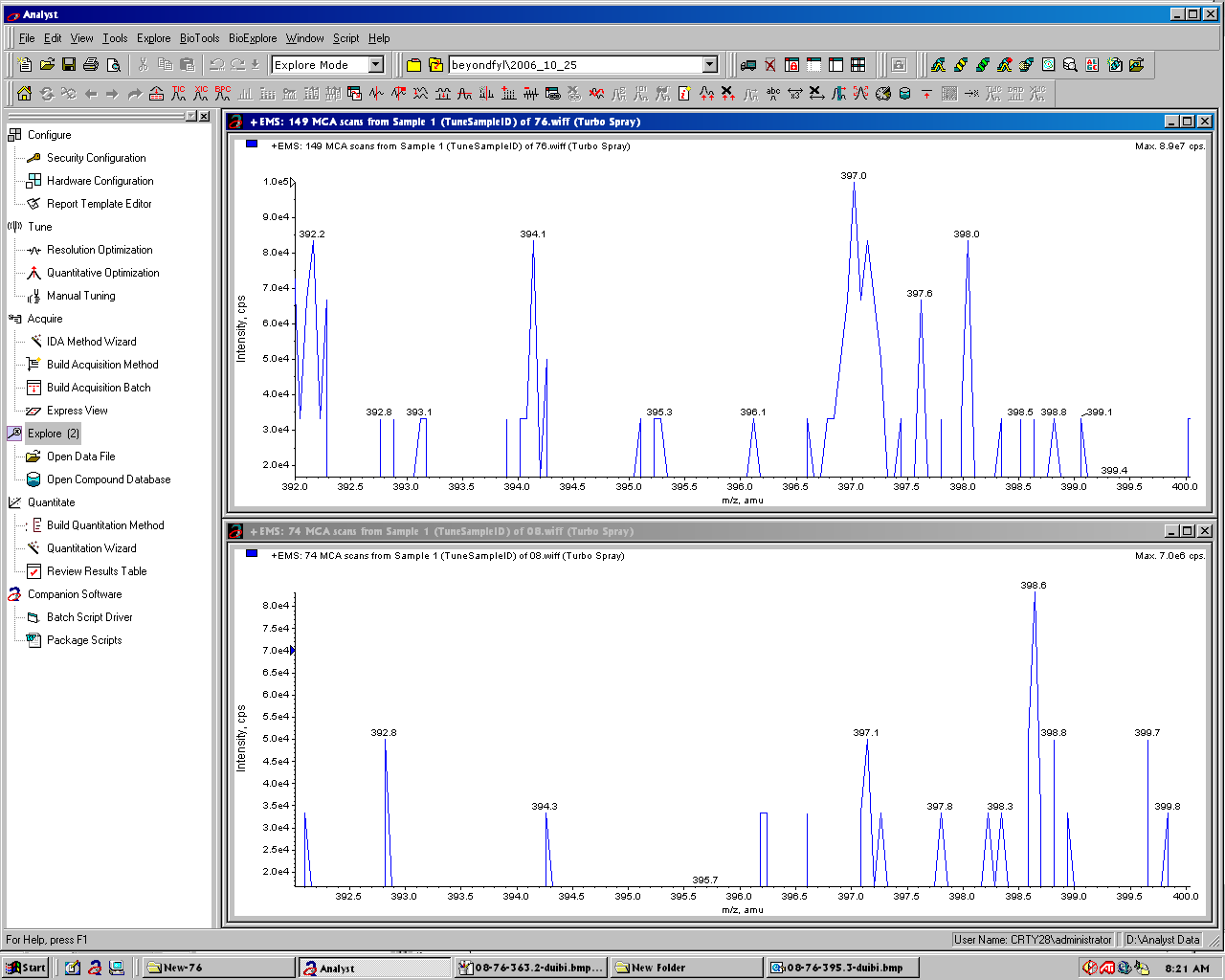

Supplement: Figure S11 — (0.12 MB TIF) [file pone.0005353.s016.tif]

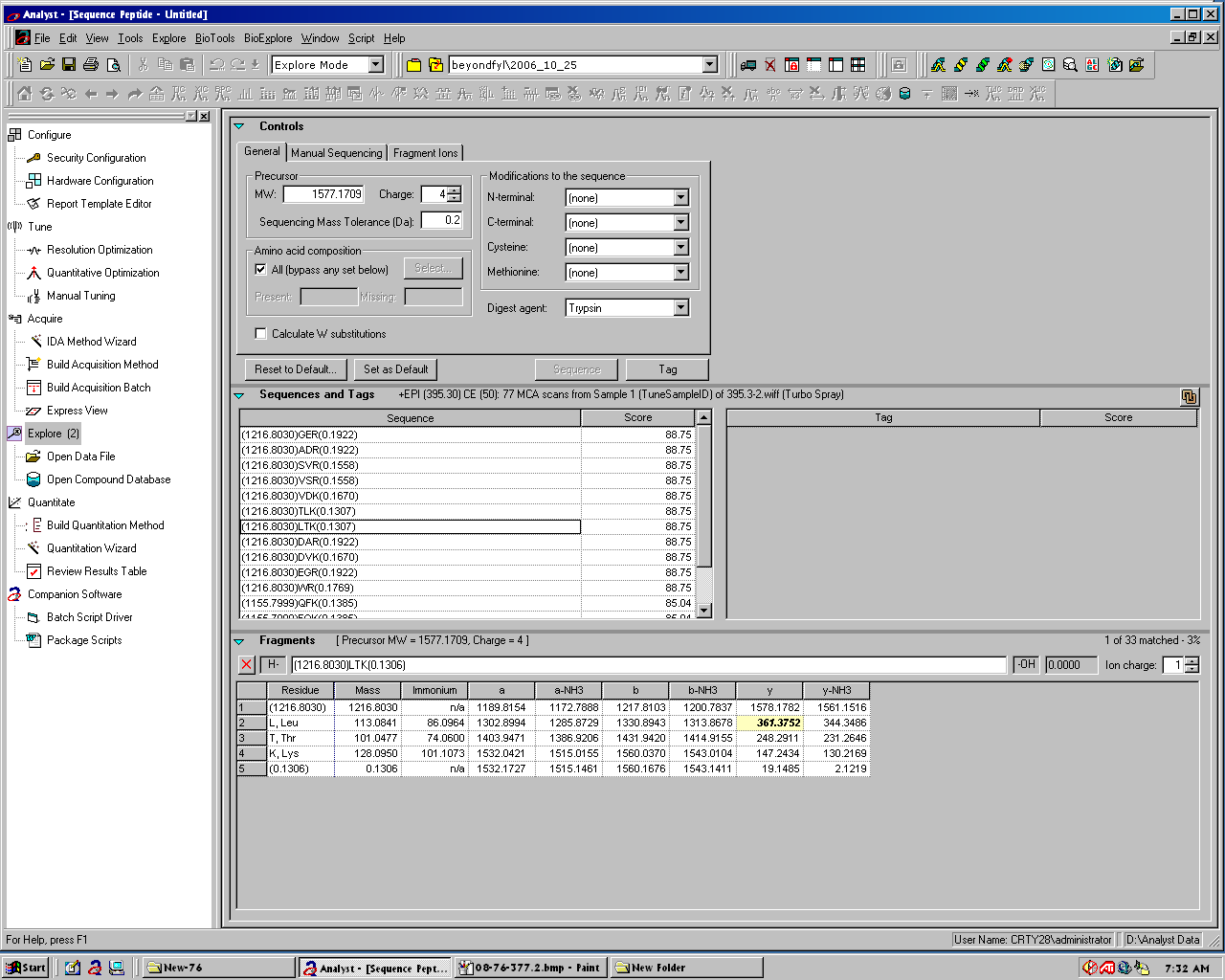

Supplement: Figure S12 — (0.12 MB TIF) [file pone.0005353.s017.tif]

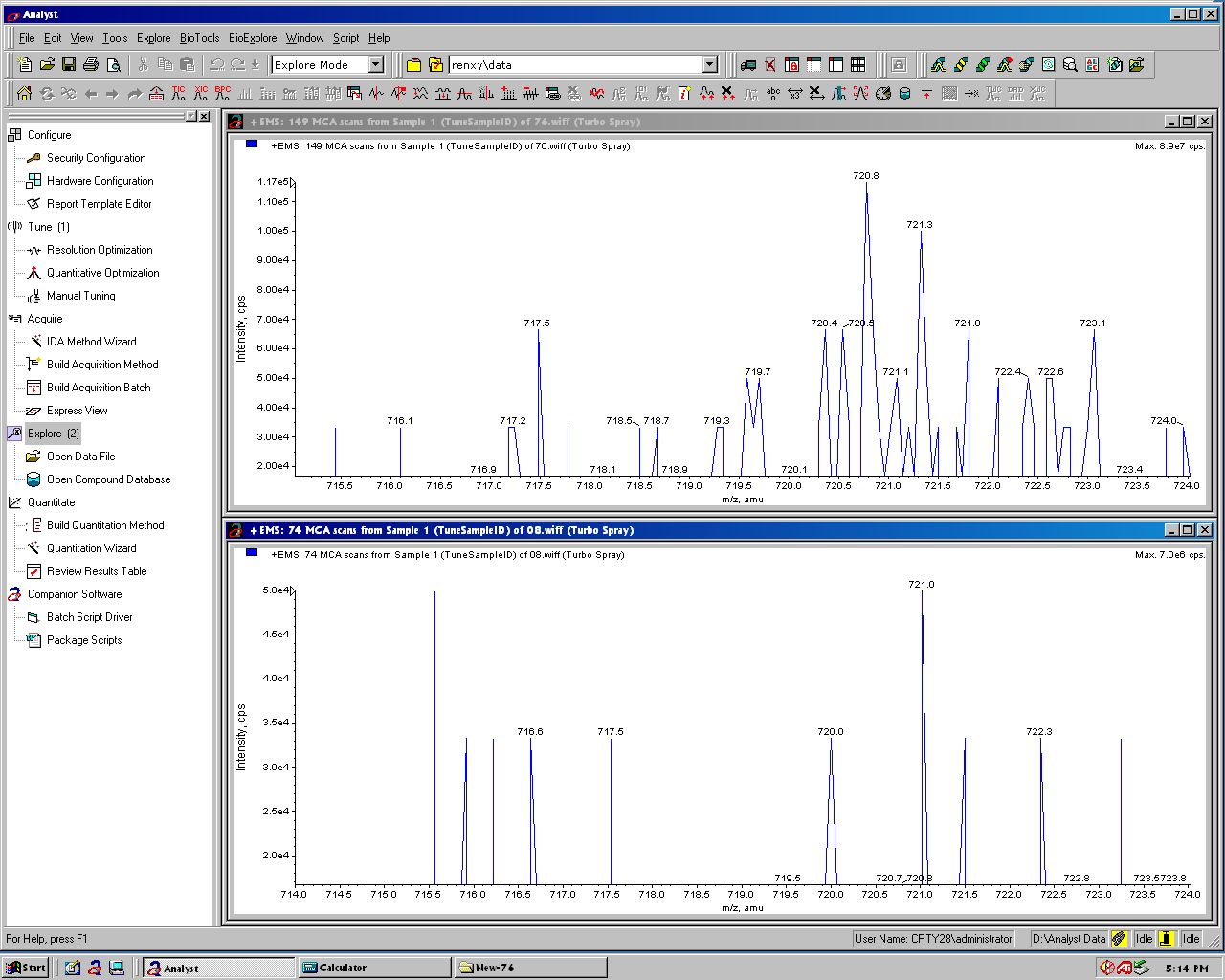

Supplement: Figure S13 — (0.12 MB TIF) [file pone.0005353.s018.tif]

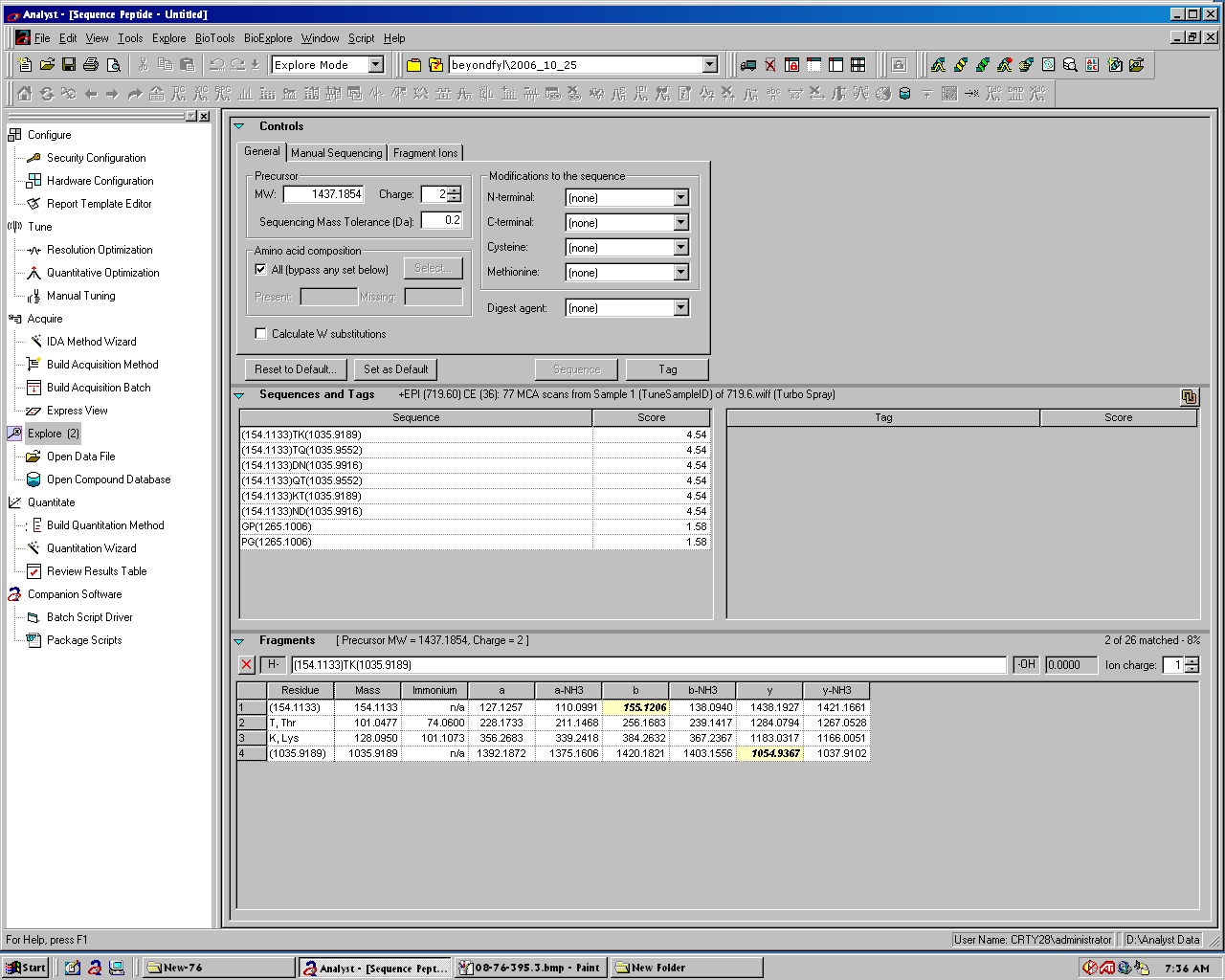

Supplement: Figure S14 — (0.12 MB TIF) [file pone.0005353.s019.tif]

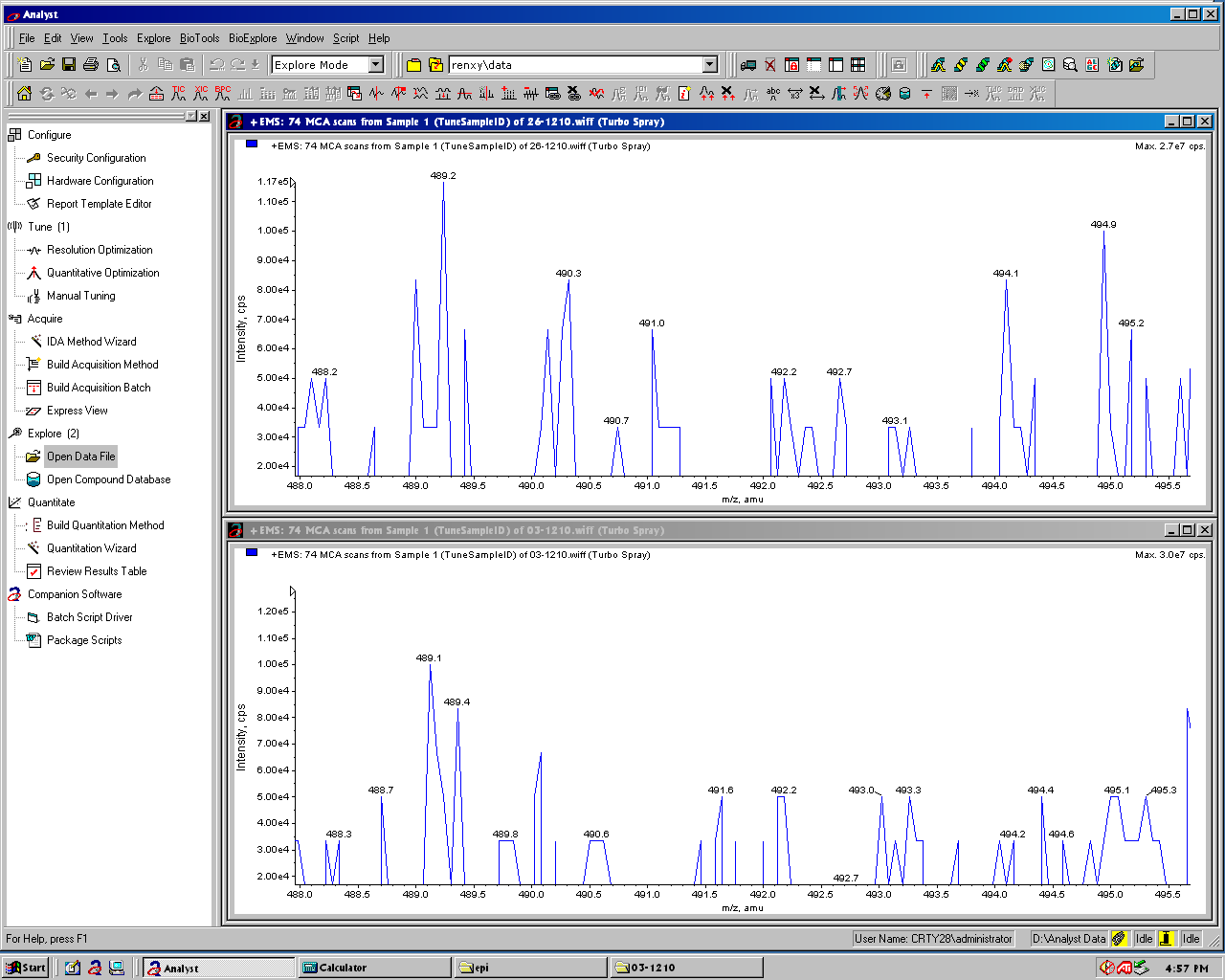

Supplement: Figure S15 — (0.13 MB TIF) [file pone.0005353.s020.tif]

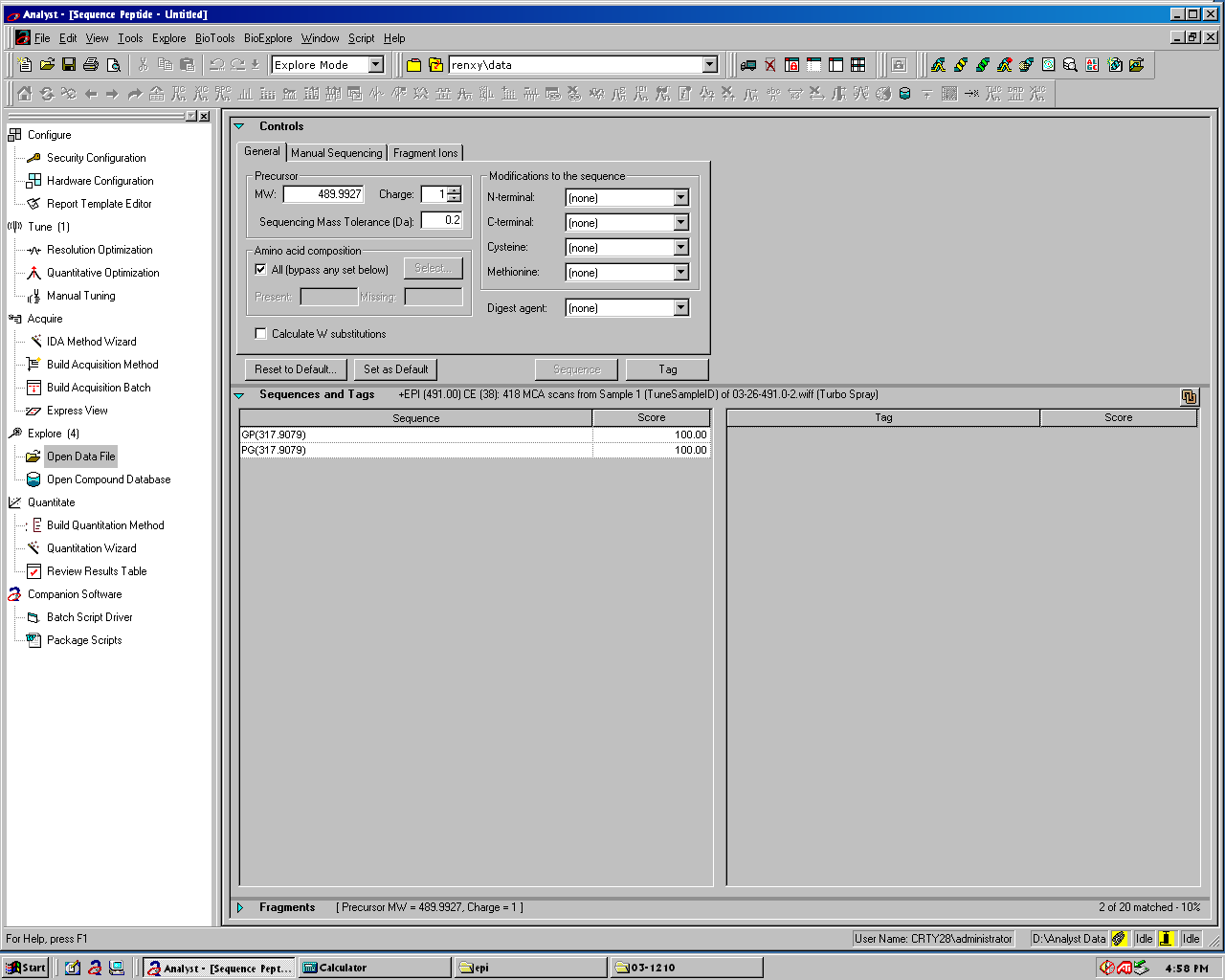

Supplement: Figure S16 — (0.10 MB TIF) [file pone.0005353.s021.tif]

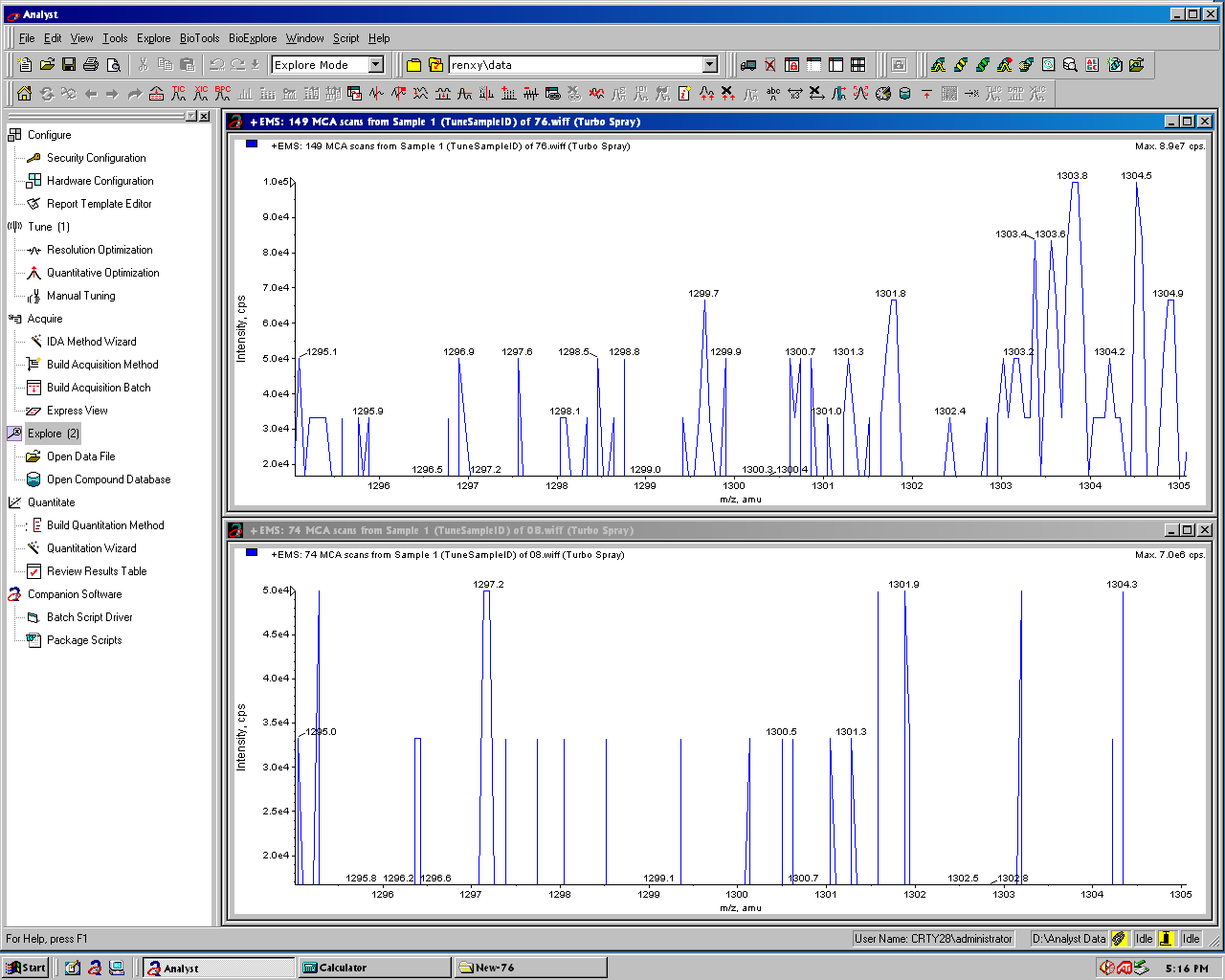

Supplement: Figure S17 — (0.13 MB TIF) [file pone.0005353.s022.tif]

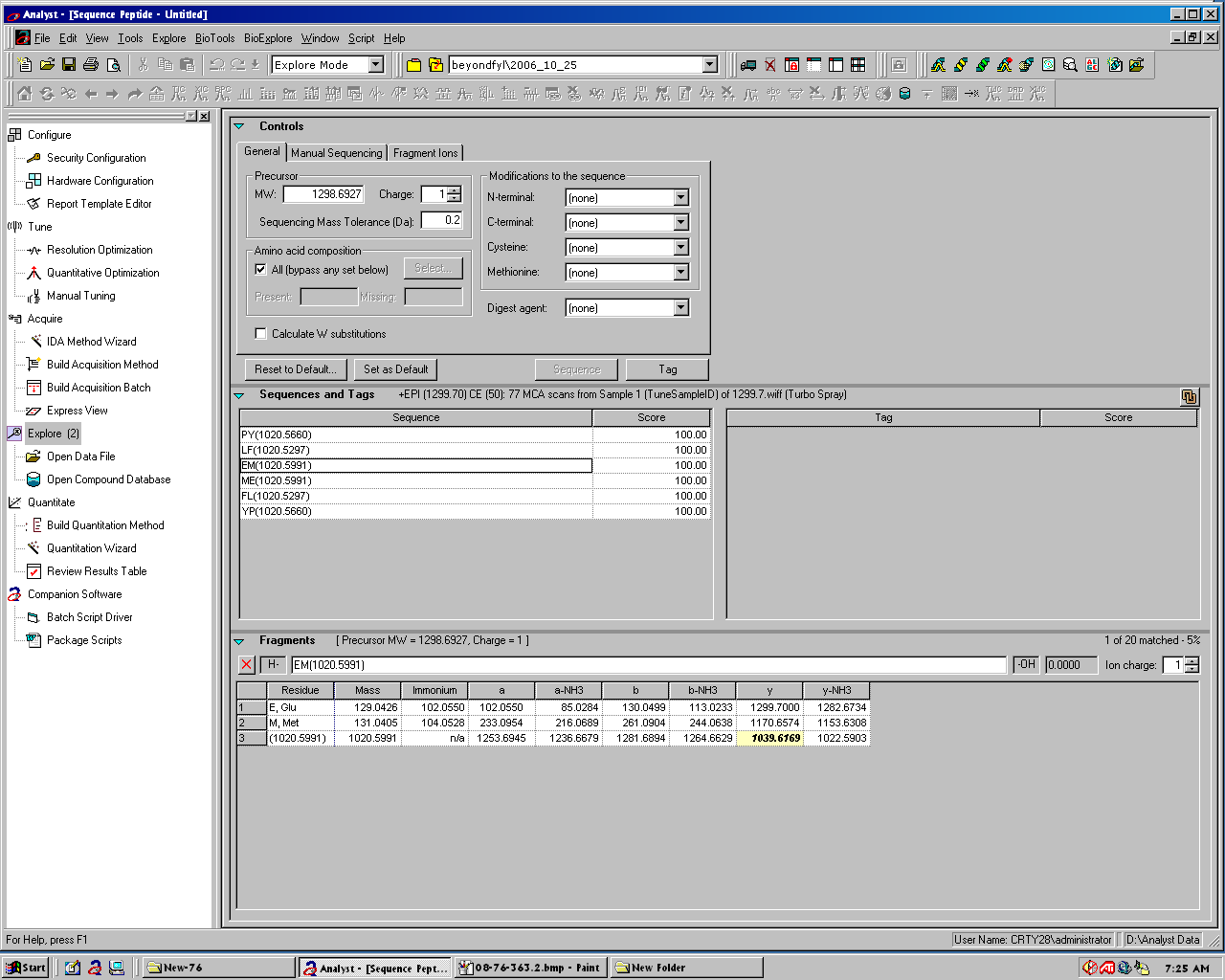

Supplement: Figure S18 — (0.11 MB TIF) [file pone.0005353.s023.tif]

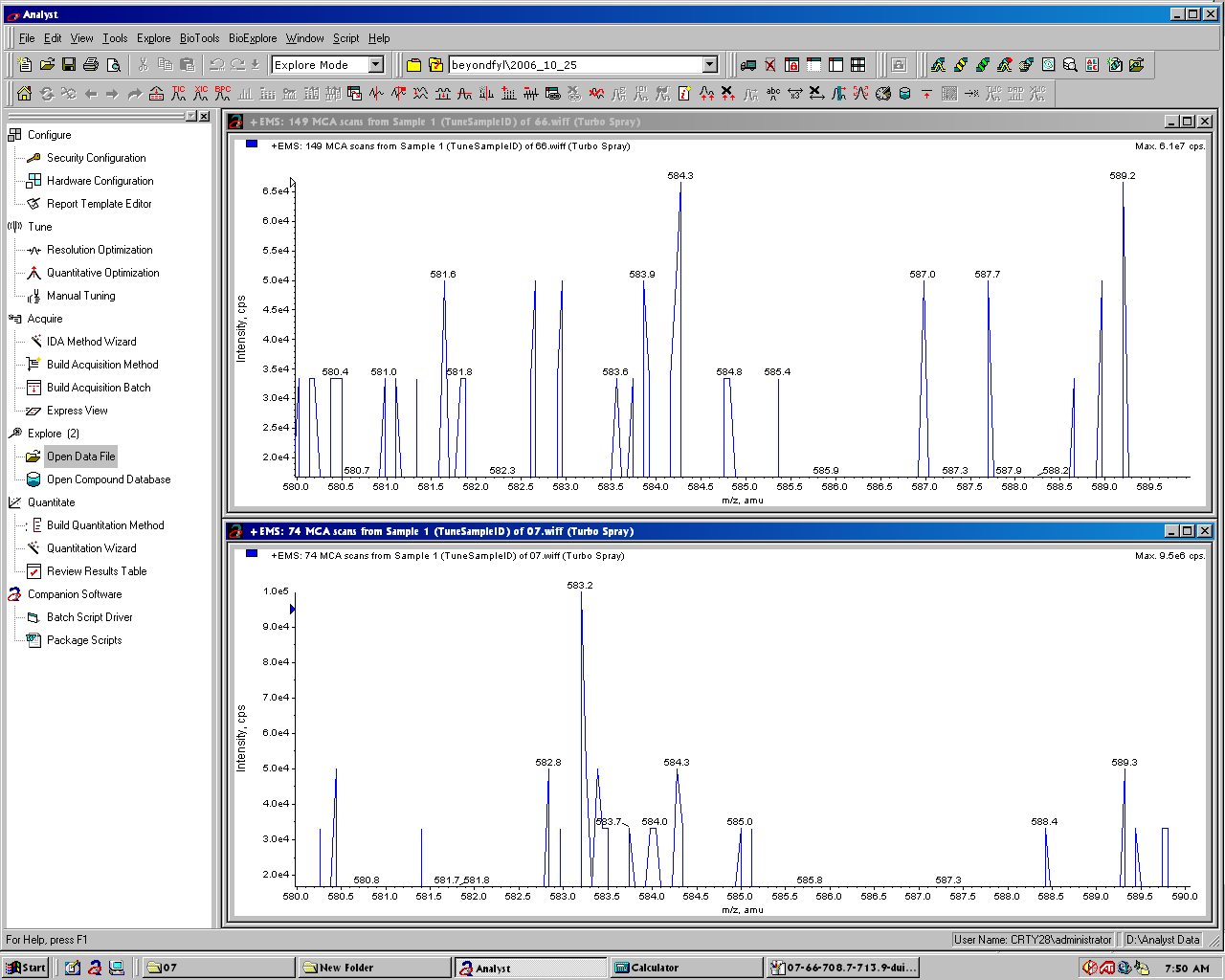

Supplement: Figure S19 — (0.12 MB TIF) [file pone.0005353.s024.tif]

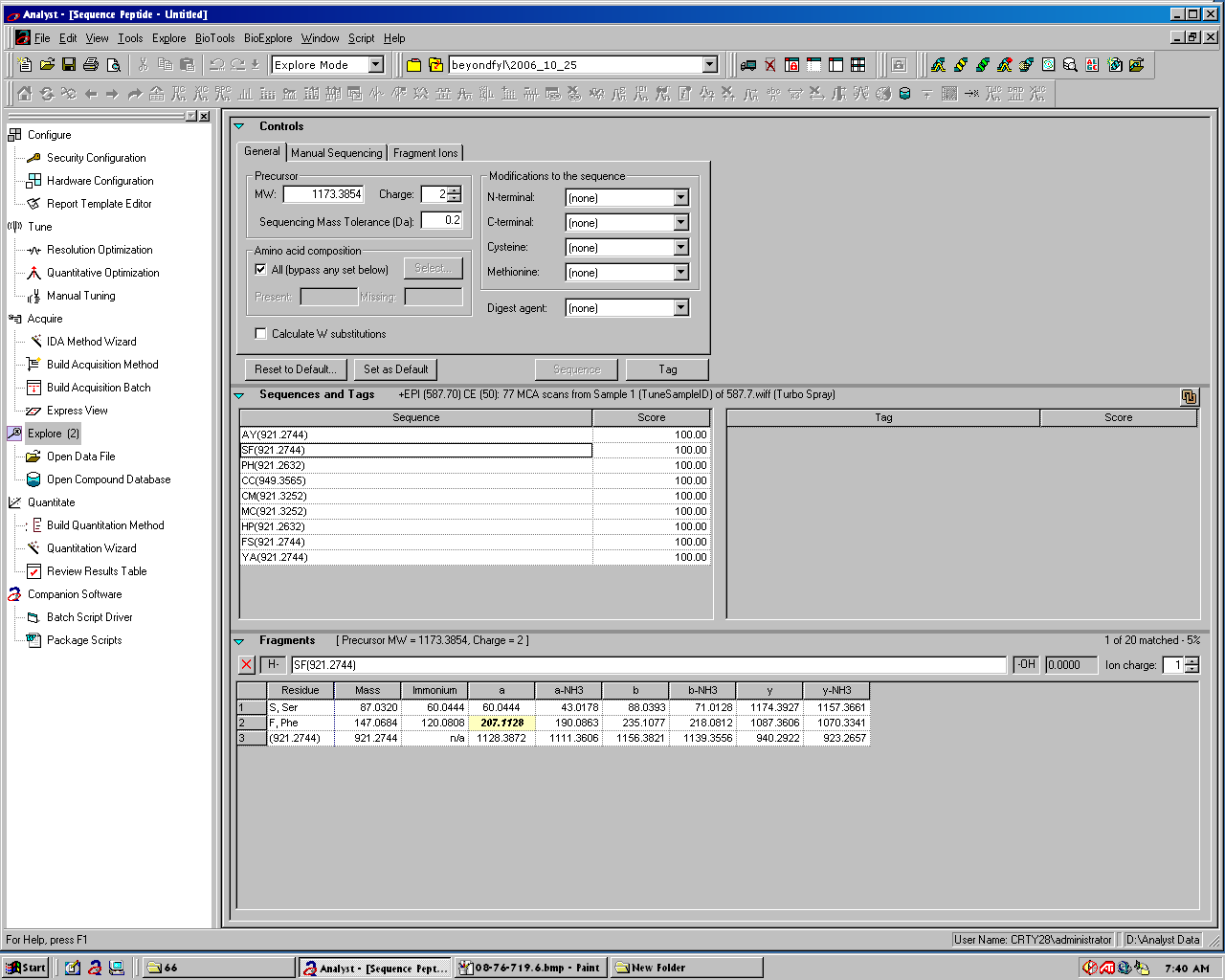

Supplement: Figure S20 — (0.11 MB TIF) [file pone.0005353.s025.tif]

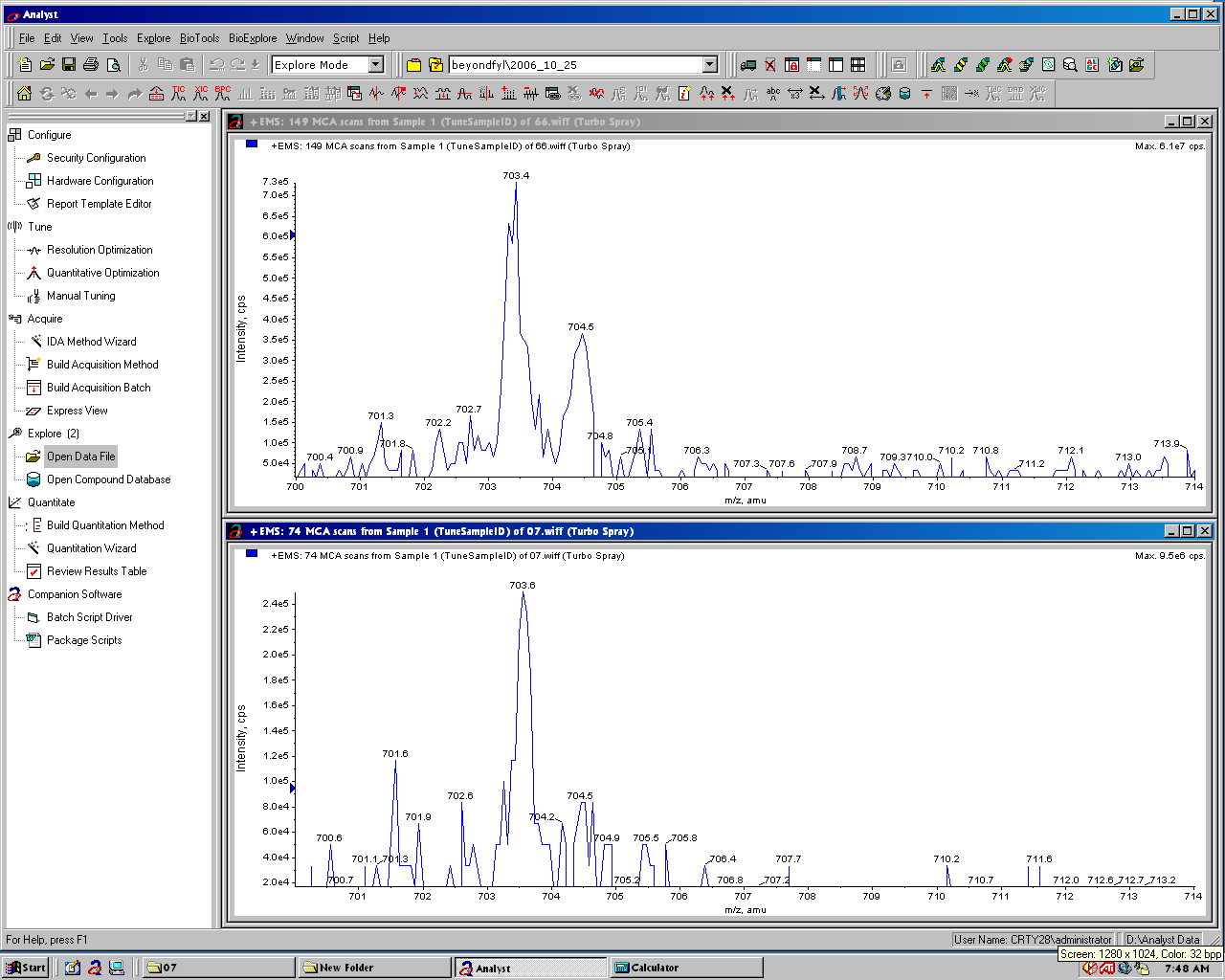

Supplement: Figure S21 — (0.12 MB TIF) [file pone.0005353.s026.tif]

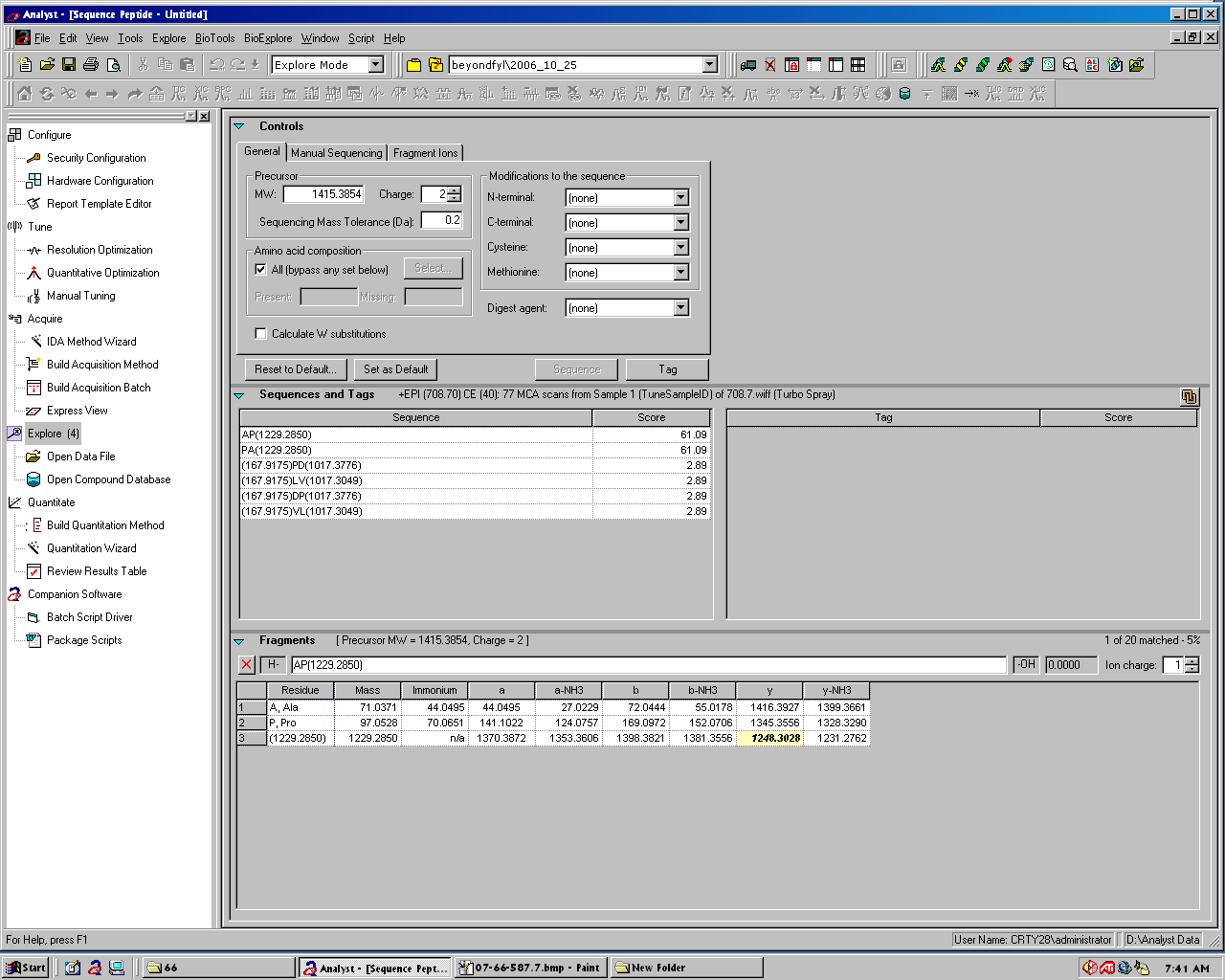

Supplement: Figure S22 — (0.11 MB TIF) [file pone.0005353.s027.tif]

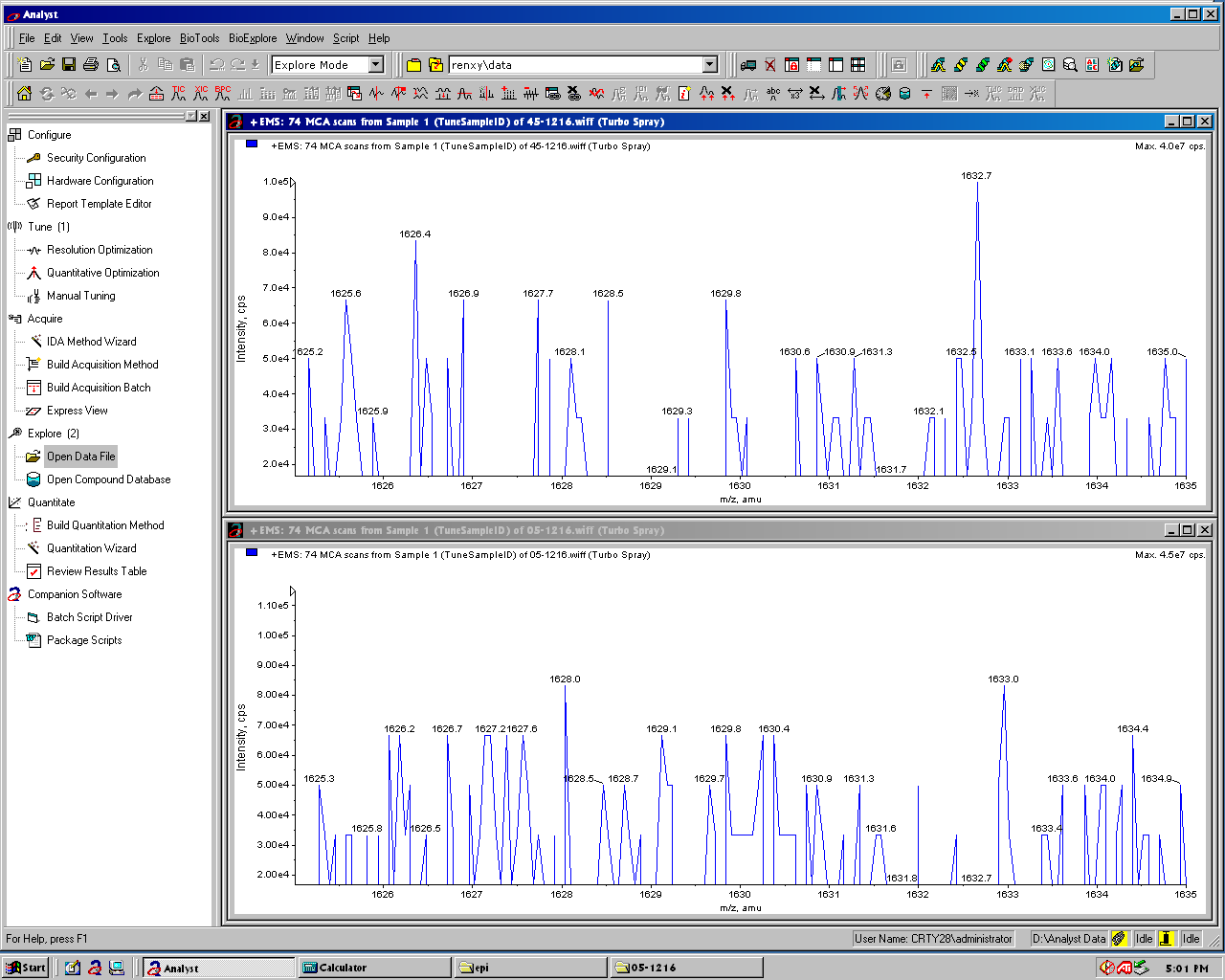

Supplement: Figure S23 — (0.13 MB TIF) [file pone.0005353.s028.tif]

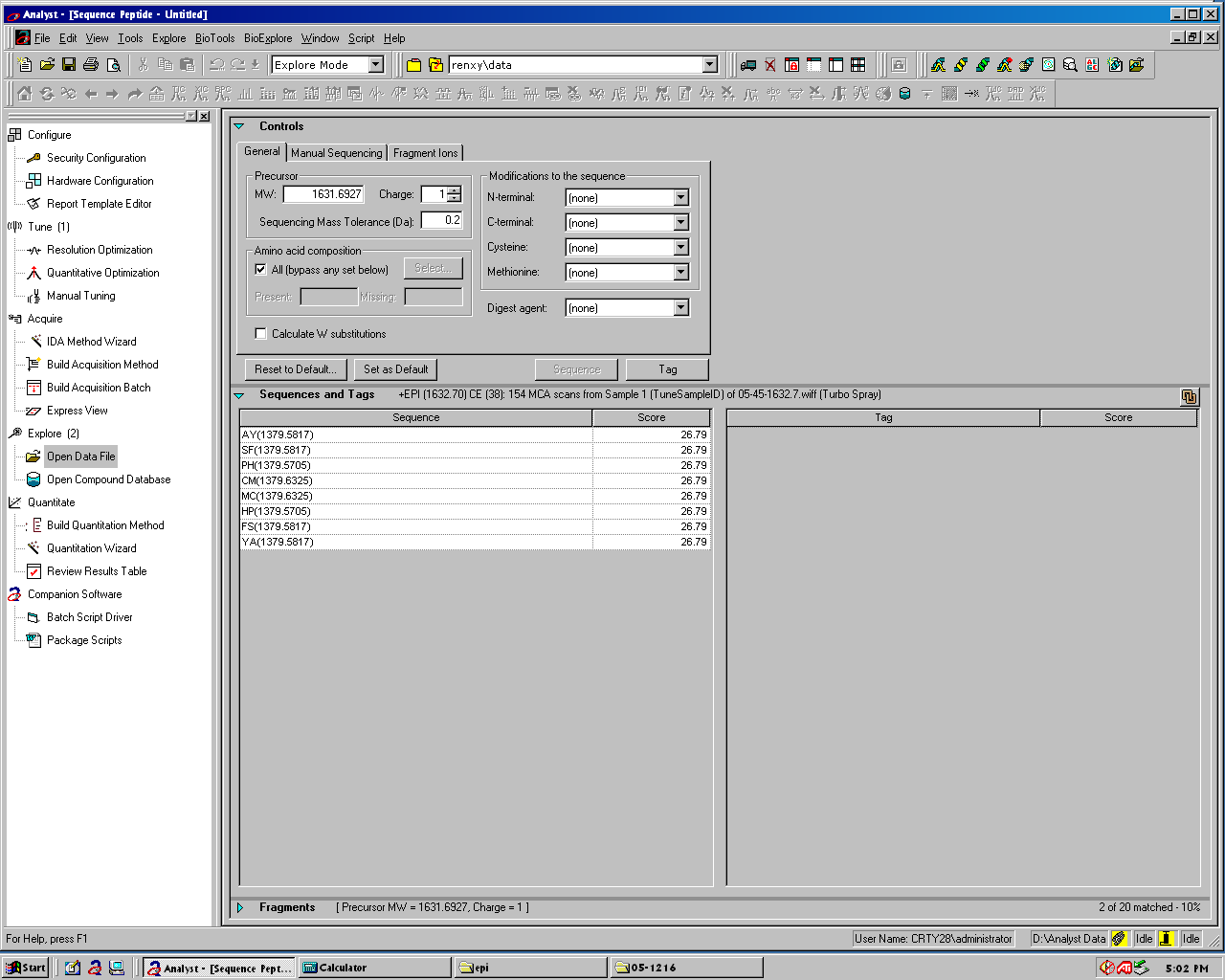

Supplement: Figure S24 — (0.11 MB TIF) [file pone.0005353.s029.tif]

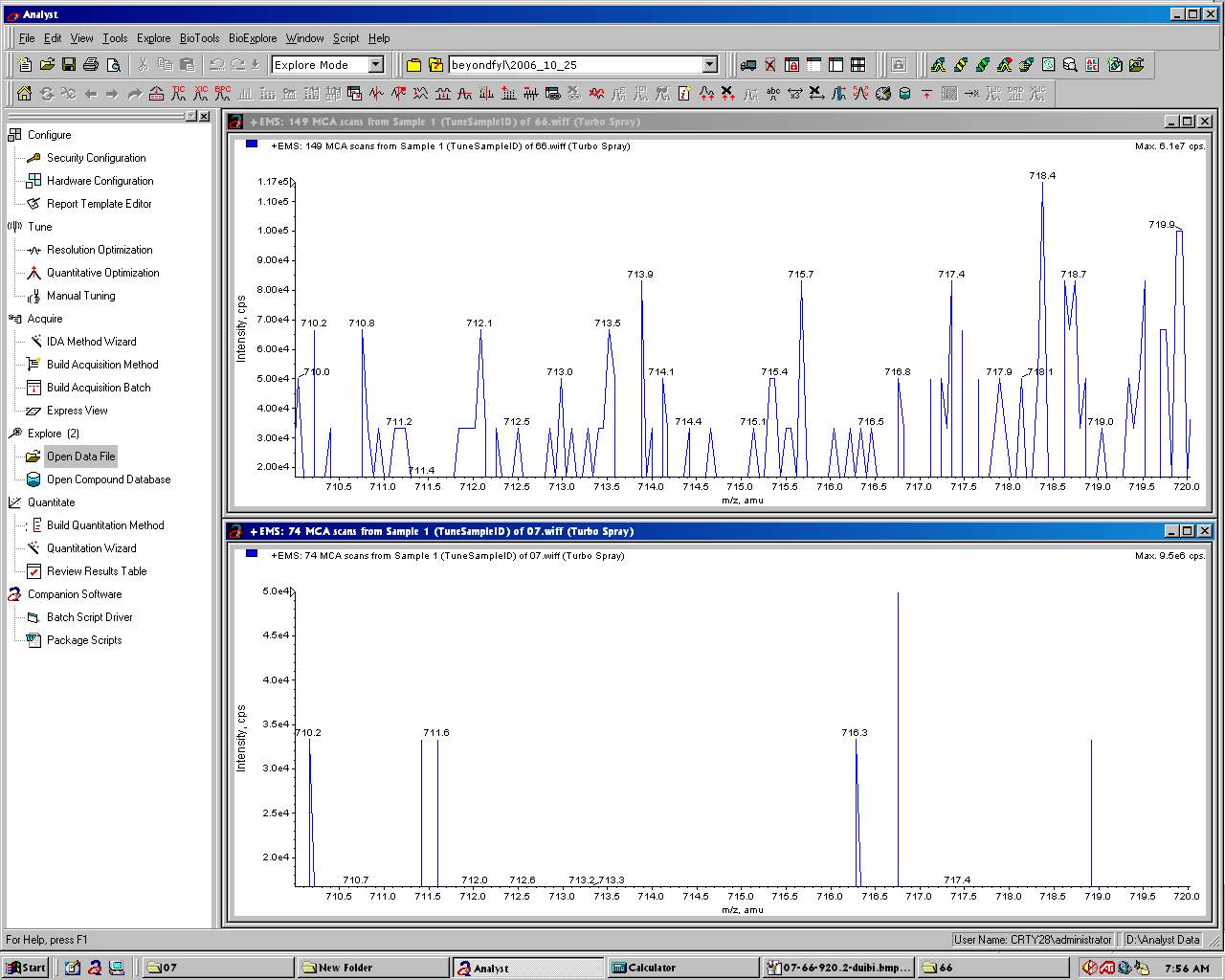

Supplement: Figure S25 — (0.13 MB TIF) [file pone.0005353.s030.tif]

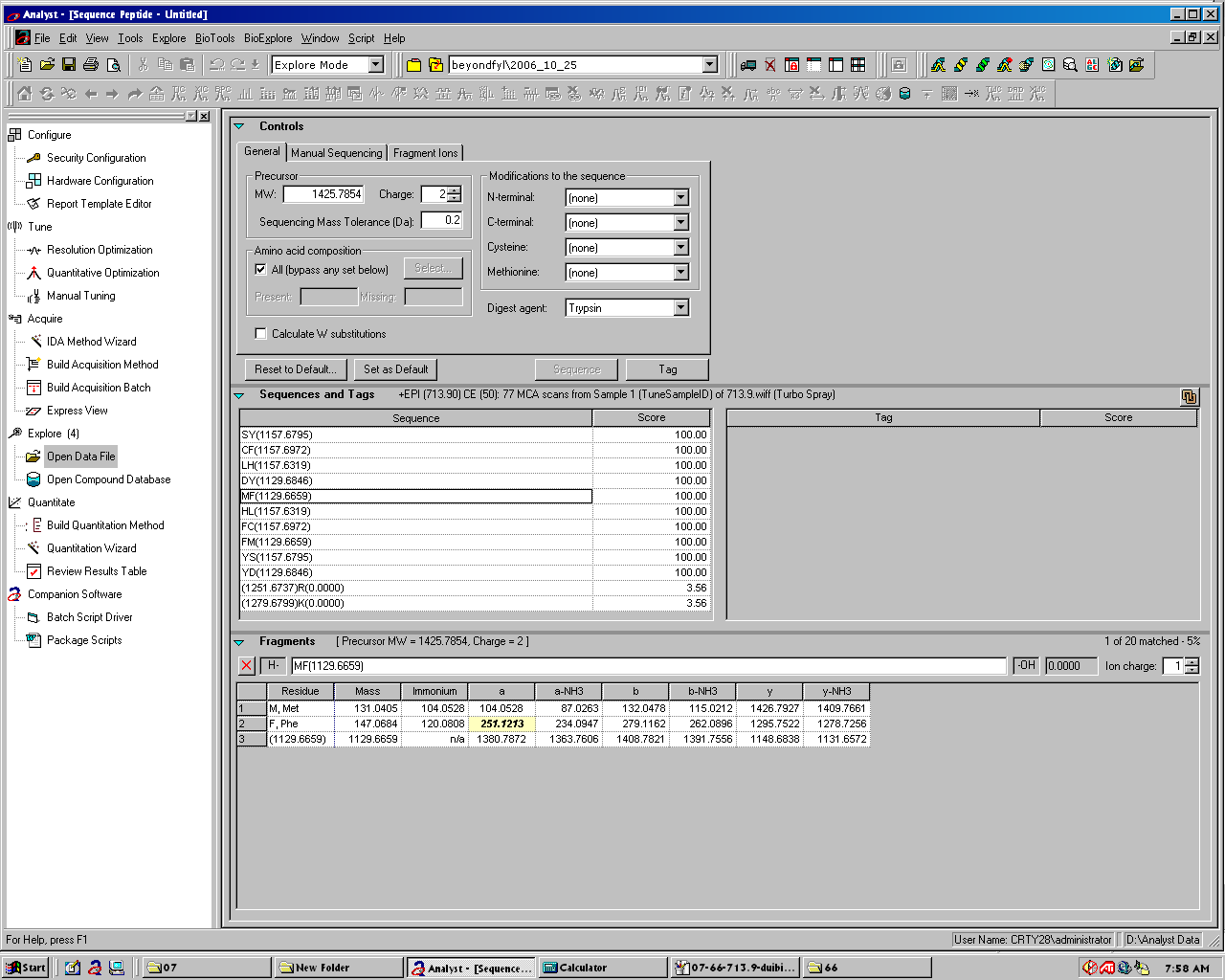

Supplement: Figure S26 — (0.12 MB TIF) [file pone.0005353.s031.tif]

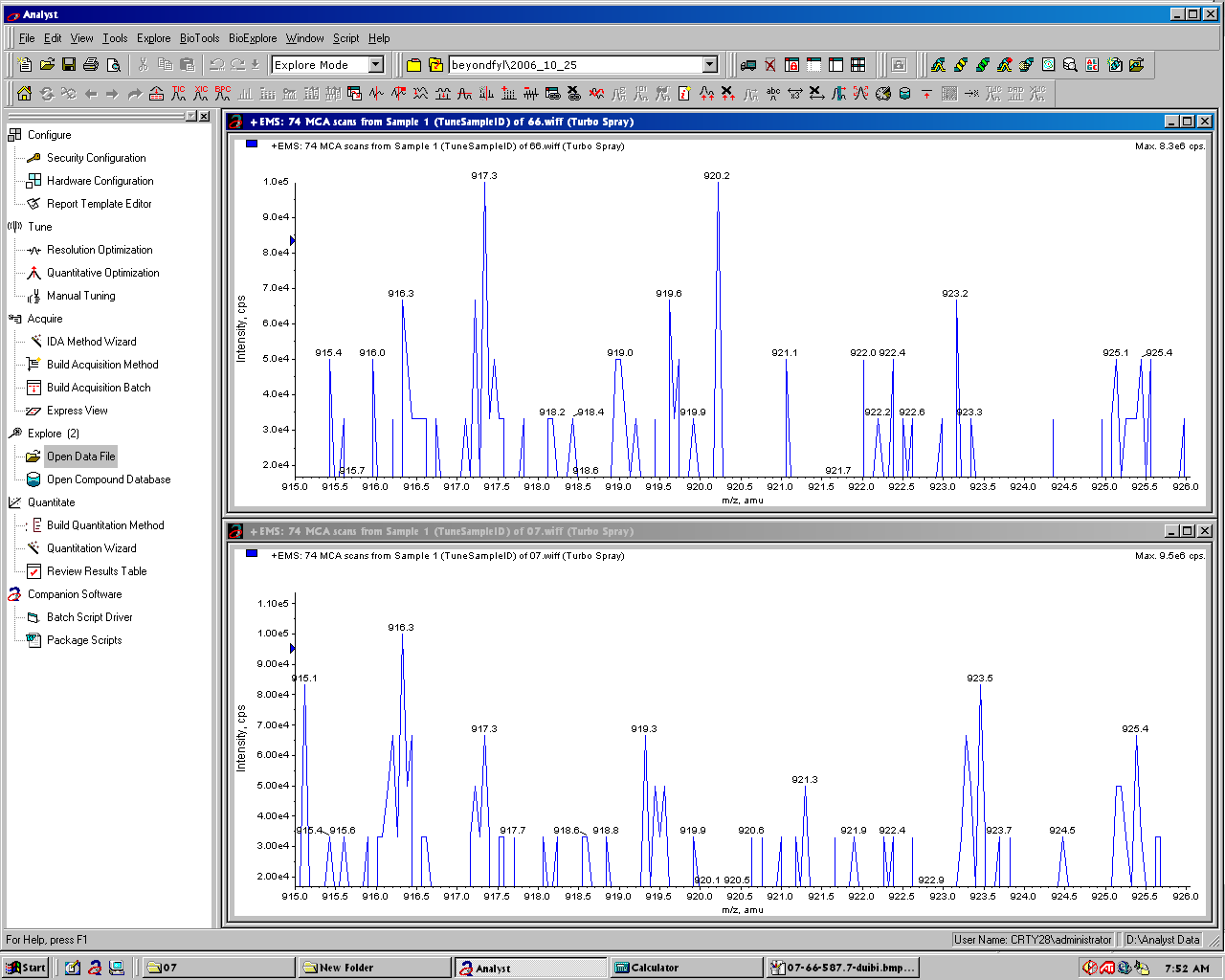

Supplement: Figure S27 — (0.13 MB TIF) [file pone.0005353.s032.tif]

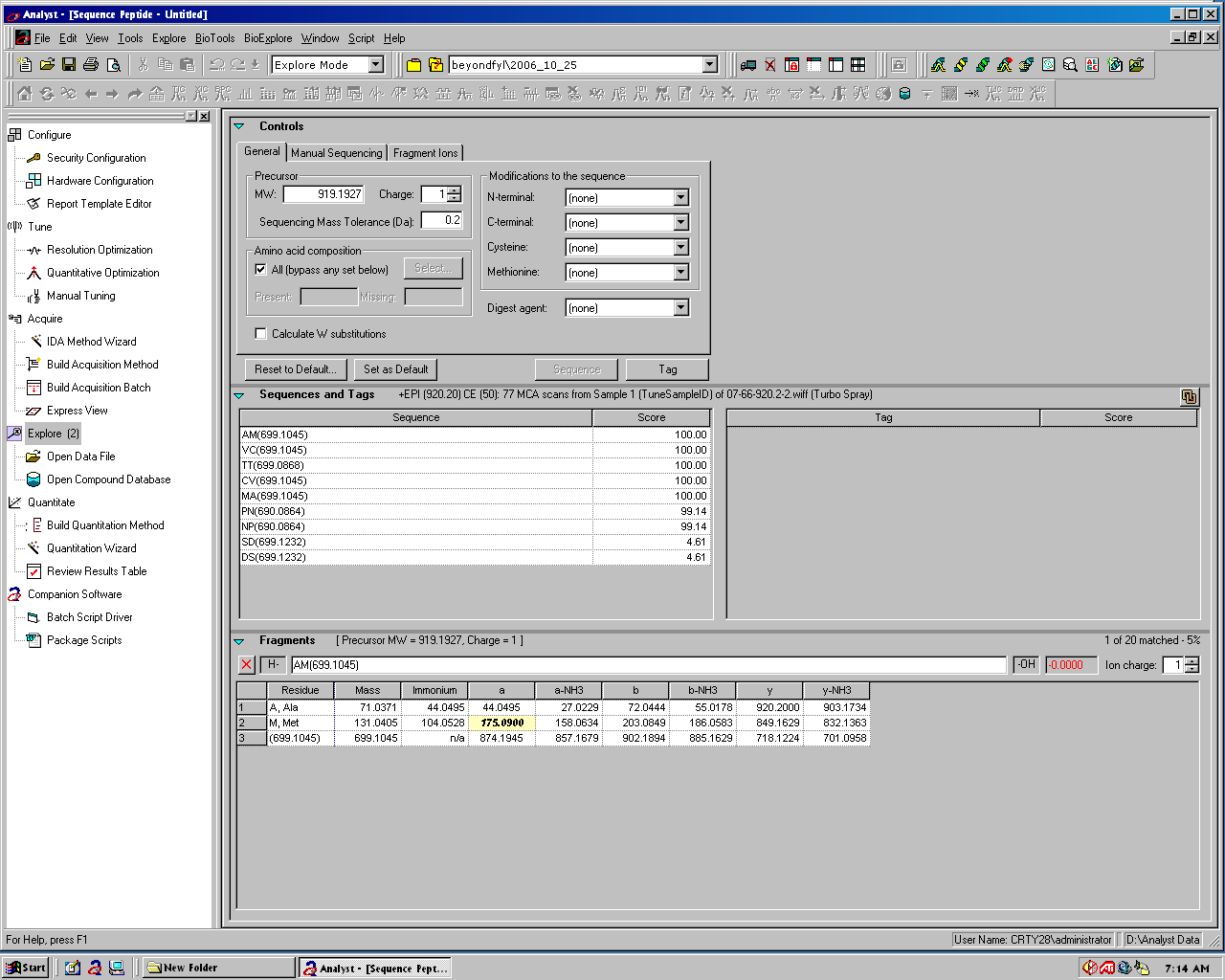

Supplement: Figure S28 — (0.11 MB TIF) [file pone.0005353.s033.tif]
